# Towards conceptual convergence: A systematic review of psychological resilience in family caregivers of persons living with chronic neurological conditions

**DOI:** 10.1111/hex.13374

**Published:** 2021-10-22

**Authors:** Odessa McKenna, Afolasade Fakolade, Katherine Cardwell, Nigèle Langlois, Karen Jiang, Lara A. Pilutti

**Affiliations:** ^1^ Interdisciplinary School of Health Sciences, Faculty of Health Sciences University of Ottawa Ottawa Canada; ^2^ School of Rehabilitation Therapy Queen's University Kingston Canada; ^3^ Health Sciences Library University of Ottawa Ottawa Canada; ^4^ Faculty of Health Sciences McMaster University Hamilton Canada; ^5^ Brain and Mind Research Institute, Faculty of Medicine University of Ottawa Ottawa Canada

**Keywords:** chronic neurological conditions, dementia, family caregivers, resilience, systematic review

## Abstract

**Background:**

The demand for family caregiving in persons with chronic neurological conditions (CNCs) is increasing. Psychological resilience may empower and protect caregivers in their role. Thus, a synthesis of resilience evidence within this specific population is warranted.

**Aim:**

In this systematic review we aimed to: (1) examine the origins and conceptualizations of resilience; (2) summarize current resilience measurement tools; and (3) synthesize correlates, predictors and outcomes of resilience in family caregivers of persons with CNCs.

**Design:**

We sourced English articles published up to July 2020 across five databases using search terms involving CNCs, family caregivers and resilience.

**Results:**

A total of 50 studies were retained. Nearly half (44%) of the studies used trait‐based resilience definitions, while about one third (36%) used process‐based definitions. Twelve different resilience scales were used, revealing mostly moderate to high‐resilience levels. Findings confirmed that resilience is related to multiple indicators of healthy functioning (e.g., quality of life, social support, positive coping), as it buffers against negative outcomes of burden and distress. Discordance relating to the interaction between resilience and demographic, sociocultural and environmental factors was apparent.

**Conclusions:**

Incongruity remains with respect to how resilience is defined and assessed, despite consistent definitional concepts of healthy adaptation and equilibrium. The array of implications of resilience for well‐being confirms the potential for resilience to be leveraged within caregiver health promotion initiatives via policy and practice.

**Patient or Public Contribution:**

The findings may inform future recommendations for researchers and practitioners to develop high‐quality resilience‐building interventions and programmes to better mobilize and support this vulnerable group.

## 1 INTRODUCTION

Chronic neurological conditions (CNCs) represent the leading cause of disability and the second most common cause of death worldwide.^1^ Globally, it is estimated that approximately one billion people, roughly one in six of the world's total population, are currently living with a CNC.^1^ Depending on their origin and aetiology, CNCs are typically divided into four groups: (1) sudden‐onset conditions (e.g., acquired brain injury [ABI], spinal cord injury [SCI], traumatic brain injury [TBI]); (2) intermittent conditions (e.g., epilepsy); (3) progressive conditions (e.g., dementia, multiple sclerosis [MS], Parkinson's disease [PD], motor neuron disease [MND] and other neurodegenerative disorders); and (4) stable with/without age‐related degeneration (e.g., polio or cerebral palsy).^2^

CNCs have an enduring time course and are associated with various complex symptoms, including cognitive impairments, behavioural and psychological problems and marked physical deficits.^2^, ^3^ Neurological symptoms and their accompanying disability present challenges for the individual, as independence, functioning and the ability to manage life roles (e.g., employment) are limited. For instance, studies of progressive CNCs (e.g., MS and PD) have reported that the challenges associated with disability management contribute to increased unemployment rates.^4^ These findings reflect limitations in the ability to perform occupational and social roles within affected populations.^4^, ^5^, ^6^

CNC‐related disability results in many persons with these conditions requiring support from others to carry out in‐home tasks of everyday living.^7^, ^8^, ^9^, ^10^ This role is typically fulfilled by an informal caregiver—an individual responsible for providing unpaid care for family members or close friends.^11^ Caregivers often experience role overload, financial strain and are unequipped to provide complex support for their care recipients.^8^, ^12^ The extent of this ongoing commitment can culminate in adverse mental and physical health outcomes.^12^, ^13^ For family caregivers of persons with CNCs, the caregiving role may contribute to increased stress, depression, anxiety, social isolation and poorer reported quality of life in comparison to the general noncaregiving population.^13^, ^14^, ^15^, ^16^ This phenomenon is referred to as *caregiver burden*.^17^

Indeed, depleted caregiver well‐being, or burden, impacts the caregiver's ability to provide sufficient support, and is further linked to increased rates of institutionalization of people living with CNCs.^18^ Nevertheless, the experience of caring for a loved one with a CNC is broad, dynamic and rarely uniform.^19^ Despite facing difficulties, some caregivers experience fewer caregiving consequences, and report rewarding and fulfilling aspects of providing care (e.g., personal growth, strengthening of relationships, enhanced compassion)^20^, ^21^ and positive health outcomes (e.g., reduced depressive symptoms).^22^, ^23^ Such variability in experience suggests that not all caregivers are harrowed by burden, and that certain caregivers are better equipped to succeed in their role than others. Thus, further exploration of protective strategies that may buffer against the negative effects of burden is needed, and this review seeks to address this gap in knowledge.

To account for this variability, research in the caregiving field is becoming increasingly focused on a protective construct—resilience—which, when described briefly, denotes caregivers' ability to adapt to the physical and psychological requirements of their role.^24^, ^25^ This transition echoes a paradigm shift in research from a burden‐centred caregiving model to a strengths‐based model that fixates on healthy development in spite of health risks.^26^, ^27^ Still, there remains ample debate in the literature regarding how psychological resilience is defined. Traditionally, *trait definitions* are used to conceptualize resilience, whereby researchers illustrate resilience as a fixed personal attribute or inherent ability.^28^, ^29^, ^30^ This distinction suggests that resilience is stable and unmalleable across the life span.^30^ More recently, scholars have investigated the adaptive mechanisms underlying resilience, conceptualizing resilience as a dynamic *process*.^29^ Defining resilience as an adaptive process accepts that resilience may fluctuate in the face of different challenges and stages of the life course and, in turn, is modifiable.^30^, ^31^ To further apprise the debate encircling resilience, Windle^29^ conducted an extensive review of over 270 resilience‐related studies, generating the following definition: ‘Resilience is the process of negotiating, managing and adapting to significant sources of stress or trauma. Assets and resources within the individual, their life and environment facilitate this capacity for adaptation and “bouncing back” in the face of adversity. Across the life course, the experience of resilience will vary’.

Evaluation of interventions and policies intended to foster resilience is dependent upon reliable and validated measures. As a reflection of the ambiguity of the resilience construct, a number of resilience measures are available, with minimal progress towards a standardized measure for broad applications.^32^, ^33^, ^34^ A methodological review of 15 resilience scales determined that the Connor–Davidson Resilience Scale (CD‐RISC),^32^ the Resilience Scale for Adults (RSA)^35^ and the Brief Resilience Scale (BRS)^36^ obtained the highest ratings among authors, despite quality and psychometric deficiencies.^33^ Most scales reflect the availability of assets that contribute to resilience (e.g., CD‐RISC)^37^ or evaluate resilience as an outcome of the capacity to ‘bounce back’ (e.g., BRS).^33^, ^34^ Presently, few measures are available that account for the complexity of resilience from a multilevel and temporal perspective.^33^ With limited access to quality scales developed for use in the general adult population, researchers lack robust evidence to inform their choice of resilience measure for differing target populations and contexts.^33^

To understand caregiving challenges and the mechanisms by which resilience operates within the caregiving context, multiple studies^25^, ^38^, ^39^ have used the Ecological Model of Resilience.^31^ This model suggests that resilience operates fluidly across multiple interrelated levels including individual, community and society.^31^ This model identifies resources and assets, existent within each of these levels, that may enhance caregiver risk or, alternatively, act to foster resilience.^31^ More recently, O'Dwyer et al.^40^ proposed a model of resilience in caregivers that conceptualizes resilience as a cyclical process, accounting for the subjective experience of adversity, with varying progressions and magnitudes.

Dissonance persists in the resilience and caregiving literature. A qualitative study among dementia caregivers found that caregivers did not agree on whether resilience was a trait or process, nor could they concur on the factors associated with resilience and its causal pathways.^40^ Similarly, a systematic review outlined mainly individual factors as major components of resilience among dementia caregivers; however, the authors acknowledged that there is no single avenue to increase resilience.^41^ A recent systematic review determined that resilience was associated with improved caregiver quality of life and alleviated caregiver burden in end‐of‐life and palliative care contexts, although the authors observed a lack of interest in other psychological aspects that may contribute to resilience.^42^

Although the literature supports the notion that a broad range of factors may influence caregiver resilience, the lack of congruence with respect to the conceptualization and measurement of resilience within the literature presents a challenge for future research and practice.^40^ Enhancing our understanding of resilience, its measures and associated factors will delineate how resilience capacities may be leveraged and monitored clinically, via intervention, programmes and service development, to better support CNC caregivers in their role. The objective of this systematic review was to synthesize the current scientific literature on the concept of resilience in CNC family caregivers. We aimed to (1) critically examine origins, theoretical conceptualizations and definitions of resilience; (2) summarize current resilience measurement tools; and (3) synthesize correlates predictors, and outcomes of resilience.

## 2 MATERIALS AND METHODS

Our protocol was registered in the PROSPERO database (CRD42020206662). This systematic review was performed in compliance with the Preferred Reporting Items for Systematic Reviews and Meta‐Analyses (PRISMA) statement and reporting guidelines.^43^

### 2.1 Search strategy and selection

A peer‐reviewed search strategy^44^ was developed in consultation with a health sciences librarian (N. L.). Five databases (MEDLINE(R) [Ovid], Embase Classic+Embase [Ovid], PsycINFO, CINAHL [EBSCO] and Web of Science Core Collection) were searched to locate relevant articles published from inception to 27 July 2020. The databases were selected to source peer‐reviewed articles across a variety of disciplines including nursing, medicine, behavioural sciences and multidisciplinary fields. As a result of the degree of novelty of our search concepts, no limits to language or publication date were applied. Searches were limited to ‘human’, where possible. Reference lists were reviewed for additional publications. Relevant search terms were categorized into three distinct themes: CNCs, family caregivers and psychological resilience (see Appendix S1).

### 2.2 Eligibility criteria

Following a modified PICO (population, intervention, comparison, outcomes) framework,^45^ we included quantitative, qualitative or mixed‐methods studies that focused on psychological resilience among community‐dwelling adult family caregivers (≥18 years old) of adults with CNCs (see Table 1). Articles that were not available in English were excluded. We excluded meta‐analyses, dissertations, systematic reviews, case reports, opinion pieces, commentaries and grey literature.

**Table 1:** Inclusion and exclusion criteria based on a modified PICO framework^45^

PICoS	Inclusion criteria	Exclusion criteria
Population	Family caregivers of adult persons living with a CNC Community‐dwelling adults (≥18 years old)	Formal/paid caregivers Caregivers of non‐CNC or paediatric populations
Phenomenon of interest	Psychological resilience in individual family CNC caregivers	Resilience at the dyadic or community level Proxy or composite measures of resilience
Context	Any country Primary informal home care	Clinical or formal healthcare settings
Study type	Quantitative, qualitative, mixed‐methods original research in the English language	Secondary research Unavailable in the English language

Abbreviation: CNC, chronic neurological condition; PICO, population, intervention, comparison, outcomes.

### 2.3 Screening process

Retrieved articles were managed using Covidence online systematic review software (Veritas Health Innovation Ltd.). One author (N. L.) ran the initial search, and another (L. P.) merged the results into Covidence, where electronic data could be exported, tracked, deduplicated and managed. A two‐stage screening process was used to determine eligibility for inclusion. Articles were first screened for relevance by title and abstract by three reviewers (O. M., K. C., K. J.), with the intention of retaining only articles that involved resilience (i.e., resilience was directly referred to in the title or abstract). Any articles with ambiguous representations of resilience were conservatively retained to the next level of review. In the second stage, full texts were reviewed based on eligibility criteria. Agreement of two reviewers (O. M. and K. C.) was required for article inclusion at this stage, resulting in 100% interreviewer agreement. Discrepancies between reviewers were resolved by the last author (L. P.).

### 2.4 Data extraction

Data were extracted using an Excel template developed by the research team. The following parameters were extracted: (a) study information (i.e., author, year, country, purpose, design, recruitment setting and sample size); (b) participant characteristics (i.e., age, gender); (c) caregiving context variables (relationship with care recipient, CNC, participant eligibility criteria); and (d) resilience components (operationalized definition of resilience, source of definition, measure of resilience, resilience score, resilience‐related results). Two independent reviewers completed the data extraction (O. M. and K. C.). Once both reviewers completed their respective extractions, the results were compared, and any discrepancies were discussed in detail and clarified in a consensus meeting. If consensus among reviewers was not reached, the final decision was made by the last author (L. P.). The authors of three (6%) studies were contacted for missing resilience score data.^46^, ^47^, ^48^ Of those contacted, we received additional data from the authors of one study.^47^

## 3 RESULTS

**Figure 1:**
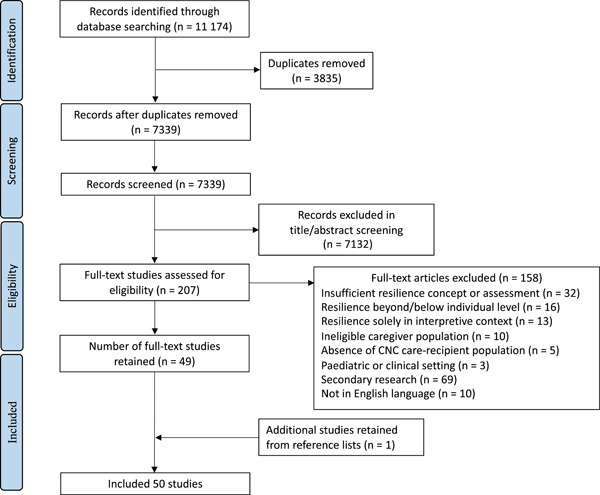
PRISMA flow diagram of the study selection process. CNC, chronic neurological condition

Following the removal of duplicates (*n* = 3835), 7339 studies remained to be screened (Figure 1). The title and abstracts of these studies were screened, and 207 articles were subjected to full‐text screening. Following our review, 49 publications fulfilled the inclusion criteria. An additional publication was located by reviewing the reference lists of included articles. Thus, a total of 50 publications were retained. Articles reporting data from the same participant population at different time points are reported together.

### 3.1 Study characteristics and caregiver sample demographics

#### 3.1.1 Study characteristics

Most (*n* = 46, 92%) studies were published within the last 10 years of conducting our search (i.e., during or after the year 2010). The majority of studies originated from Europe (*n* = 20, 40%)^38^, ^47^, ^48^, ^49^, ^50^, ^51^, ^52^, ^53^, ^54^, ^55^, ^56^, ^57^, ^58^, ^59^, ^60^, ^61^, ^62^, ^63^, ^64^, ^65^ and North America (*n* = 20, 40%),^19^, ^25^, ^46^, ^66^, ^67^, ^68^, ^69^, ^70^, ^71^, ^72^, ^73^, ^74^, ^75^, ^76^, ^77^, ^78^, ^79^, ^80^, ^81^, ^82^ followed by Asia (*n* = 4, 8%),^83^, ^84^, ^85^, ^86^ South America (*n* = 4, 8%)^87^, ^88^, ^89^, ^90^ and Australia (*n* = 2, 4%).^91^, ^92^ Across studies, the sample size ranged between 18^71^ and 691.^80^, ^81^ Most of the quantitative studies were cross‐sectional (*n* = 34, 68%) or longitudinal designs (*n* = 1, 2%). Only four studies (8%) were intervention‐based.^70^, ^75^, ^84^, ^85^ Seven studies (14%) used a qualitative design involving semi‐structured interviews,^38^, ^52^, ^53^, ^73^, ^74^ open‐ended questionnaires^19^ or content analysis.^25^ Four (8%) studies adopted a mixed‐methods design.^54^, ^71^, ^72^, ^86^

#### 3.1.2 Caregiver demographics

A total of 5992 caregivers were sampled across studies. As shown in Table 2, the mean age of the caregivers ranged between 40^77^ and 76^78^ years. Most caregivers were women (55%–97%).^68^, ^87^ The majority of the caregivers were cohabitating spouses/partners (*n* = 2898, 48%), followed by offspring or children (*n* = 1674, 28%), parents (*n* = 353, 6%), siblings (*n* = 167, 3%), grandchildren (*n* = 58, 1%) or undisclosed ‘other’ (*n* = 838, 14%).

**Table 2:** Study and caregiver sample characteristics in the 50 studies included in the review

Author (year)	Country	Sample size (*n*)	Age, mean (SD)	Gender (% F)	Relationship to care‐recipient (%)	CNC
Castellano‐Tejedor and Lusilla‐Palacios (2017)^58^	Spain	75	48.55 (12.55)	84.0	Spouse/partner: 44 Offspring: 39 Sibling: 8 Parent: 5 Other: 3	SCI
Senturk et al. (2018)^59^	Turkey	103	56.5 (9.91)	85.4	Spouse: 36.9 Mother: 42.7 Father: 16.5 Relative: 3.9	Dementia
Garity (1997)^76^	USA	76	61.5 (14.1)	71.0	Spouse: 43 Offspring: 42 Sister: 8 Grandchild: 7	AD
Scholten et al. (2020)^60^	The Netherlands	157	55.5 (12.4)	61.8	Partner: 78.3 Parent: 8.9 Child: 7 Other: 5.8	SCI, ABI
Brickell et al. (2020)^77^	USA	346	40.6 (9.3)	96.2	Spouse/partner: 91 Other: 9	TBI
Simpson and Jones (2013)^91^	Australia	61 (TBI: 30 SCI: 31)	ABI: 54 (12) SCI: 50 (14)	90.2	Parent: 39.4 Spouse: 54.1 Other: 6.6	TBI, SCI
Cousins et al. (2013)^61^	UK	27	NIV	74.0	Spouse/partner: 40.1 Offspring: 7.4 Sibling: 11.1 Parent: 3.7	MND
			57.56 (11.70)			
			Declined NIV			
			65.88 (10.45)			
Elnasseh et al. (2016)^62^	Spain	105	57.71 (13.35)	74.3	NR	Dementia
Ertl et al. (2019)^63^	Spain	95	51.1 (13.85)	78.0	Spouse/partner: 60 Offspring: 26.3 Sibling: 9.5 Parent: 4.2	PD
Fitzpatrick and Vacha‐Haase (2010)^78^	USA	30	76.4 (6.0)	70.0	Spouse: 100	Dementia (AD or other)
Kimura et al. (2019)^87^	Brazil	43	51.1 (15.2)	97.1	Spouse: 48.8 Offspring: 34.9 Sibling: 9.3 Other: 7	Young‐onset AD
Ruisoto et al. (2020)^64^	Spain	283	59.93 (14.56)	65.7	Offspring: 55.5 Spouse: 40.6 Other: 3.9	Dementia
Scott (2013)^46^	USA	110	63 (11)	80.2	Spouse: 36 Offspring: 59.5 Other: 4	AD
Pessotti et al. (2018)^88^	Brazil	50	54.7 (11.1)	88.0	Wives: 32 Daughters: 54	Dementia
Wilks and Vonk (2008)^79^	USA	304	63 (13.5)	77.0	Spouse: 43 Offspring: 39 Friend: 4 Other: 14	AD
Rosa et al. (2020)^90^	Brazil	106	57.9 (13.75)	79.2	Spouse: 37.7 Offspring: 52.8 Other: 9.4	AD
Chan et al. (2019)^83^	Malaysia	207	50.4 (14.5)	79.7	Spouse: 16.4	AD
					Offspring: 61.4	
					Other: 17.4	
					Unknown: 4.8	
Dias et al. (2016)^89^	Brazil	58	62.5 (13.44)	79.3	Spouse: 44.8 Offspring: 51.7 Other: 3.4	Dementia (AD, vascular dementia, mixed dementia)
Serra et al. (2018)^65^	Spain	326	59.9 (14.6)	65.7	Spouse: 55.5 Offspring: 40.6 Other: 3.9	Dementia
Sutter et al. (2016)^47^	Spain	127	57.14 (13.01)	77.2	Spouse/partner: 17.8 Offspring: 22.2 Sibling: 60	Dementia
Jones et al. (2018, 2019)^48^	UK	80	NR	73.8	Spouse: 65 Other: 35	Dementia
Jones, Killett et al. (2019)^55^; Jones, Woodward et al. (2019)^49^	UK	110	NR	66.0	Spouse: 62 Other: 38	Dementia
Wilks et al. (2011, 2018)^80^	USA	691	61^a^	79.8	Spouse: 16.7 Offspring: 51.3 Sibling: 4.4 Grandchild: 6.6 Friend: 3.8 Other: 16.9	AD
Wilks (2008)^66^; Wilks and Croom (2008)^82^	USA	229	45^a^	90.0	Spouse: 30 Offspring: 49 Friend: 8 Grandchild: 5 Other: 8	AD
Anderson et al. (2019)^92^	Australia	131	58.2 (14.3)	80.9	Spouse: 45 Parent: 44.3 Other: 10.7	TBI
Hayas et al. (2015)^50^	Spain	237	55.6 (12.4)	77.6	Spouse: 47.3 Parent: 28.3 Child: 14.8 Sibling: 7.2 Other: 2.5	ABI
Vatter et al. (2018, 2020)^56^, ^57^	UK	136	69.44 (7.62)	85.3	Married: 94.9 Cohabitating: 5.1	PD‐related dementia
Ledbetter et al. (2020)^67^	USA	312	42.3 (11.9)	80.8	Spouse/partner: 100	SCI
O'Rourke et al. (2010)^68^	Canada	105	69.59 (8.66)	55.0	Spouse: 100	AD
Rivera‐Navarro et al. (2018)^51^	Spain	326	60.1 (14.5)	67.2	Spouse: 41.4 Offspring: 52.5 Son‐/daughter‐in‐law: 2.5 Sibling: 0.9 Other: 2.5	Dementia
Tyler et al. (2020)^69^	USA	253	59.92 (14.68)	73.1	Spouse: 68.8 Parent: 21.7 Friend: 1.2 Sibling: 4.3 Cousin: 0.4 Aunt/uncle: 1.2 Other: 2.4	PD
Ghaffari et al. (2019)^84^	Iran	54	Control 43.4 (6.3) Intervention 42.6 (6.2)	Control 70.0 Intervention 88.0	Control Spouse: 20 Offspring: 80 Intervention Spouse: 12 Offspring: 88	AD
Lavretsky et al. (2010)^75^	USA	40	Control 63.3 (13.4) Intervention 60 (9.4)	Control 55.0 Intervention 75.0	Spouse: 37.5 Offspring: 62.5	AD
MacCourt et al. (2017)^70^	Canada	200	64.4^a^	79.0	Spouse: 61.9 Parent: 23 Other: 5.1	Dementia/AD
Pandya (2019)^b^^,^ ^85^	India	96/96 (C/I)	Control 52.5 (10.67) Intervention 52.68 (11.03)	Control 86.5 Intervention 81.3	Spouse: 54.6 Offspring: 23.3 Son/daughter in‐law: 22.1	AD
Maneewat et al. (2016)^86^	Thailand	150	NR	NR	NR	Dementia
Bull (2014)^71^	USA	18	64 (14.1)	67.0	Spouse: 39 Offspring: 61	Dementia
Kidd et al. (2011)^72^	USA	20	60.2^a^	85.0	Spouse: 100	Dementia
Bekhet and Avery (2018)^19^	USA	80	57.0 (15.6)	90.0	NR	Dementia
Roberts and Struckmeyer (2018)^73^	USA	33	NR	87.9	Spouse: 42.4 Parent: 48.5 Offspring: 6.1 Sibling: 3	Dementia
Han et al. (2019)^25^	USA	39	62 (7.4)	76.9	Spouse/partner: 7.7	AD and related dementias
					Offspring: 82.1	
					Other: 10.2	
Liu et al. (2020)^74^	USA	27	69.04 (10.51)	77.8	Spouse: 46.2	Dementia
					Offspring: 50	
					Sibling: 3.85	
Donnellan et al. (2015, 2017, 2019)^38^	UK	23	75 (7.46)	69.6	Spouse: 100	Dementia

Abbreviations: ABI, acquired brain injury; AD, Alzheimer's disease; CNC, chronic neurological condition; MND, motor neuron disease; NIV, noninvasive ventilation; NR, not reported; PD, Parkinson's disease; SCI, spinal cord injury; TBI, traumatic brain injury.

^a^ SD for the total sample not reported.

^b^ Pretest values reported.

#### 3.1.3 Chronic neurological condition

The most commonly reported CNCs were progressive conditions (*n* = 43, 86%). A substantial proportion (*n* = 37, 74%) of the progressive conditions studied were dementia, Alzheimer's disease or other dementias (e.g., mixed, vascular). The remaining progressive conditions were PD (*n* = 2, 4%),^63^, ^69^ PD‐related dementia (*n* = 2, 4%)^56^, ^57^ and MND (*n* = 1, 2%).^61^ Few studies (*n* = 7, 14%) included sudden‐onset conditions including SCI,^58^, ^60^, ^67^, ^91^ ABI^50^, ^60^ and TBI.^77^, ^91^, ^92^ No studies included intermittent (e.g., epilepsy) or stable (e.g., polio, cerebral palsy) conditions.

### 3.2 Conceptualization, measurement and levels of resilience

#### 3.2.1 Resilience conceptualizations

Certain (*n* = 7, 14%) studies minimally or unclearly defined resilience,^75^, ^85^ briefly presenting it as a general protective psychological factor,^51^, ^60^ or simply in relation to stress^56^, ^57^ or positive coping (see Table 3).^47^ The remaining articles (*n* = 43, 86%) offered some type of a theoretical definition of resilience. When broadly discussed, the vast majority of included articles incorporated the idea of healthy adaptation into their conceptualizations of resilience. Further, most referred to preserving some level of well‐being, equilibrium or positive functioning in the face of adversity. Many (*n* = 13, 26%) studies referred to the significance of internal and external resources, protective factors, and relational and situational contexts in facilitating resilience development.^38^, ^48^, ^52^, ^53^, ^54^, ^55^, ^59^, ^65^, ^67^, ^74^, ^78^, ^82^, ^92^ For instance, self‐compassion was an internal resource conceptually linked to resilience.^54^

**Table 3:** Operationalized definitions of resilience by article included in the review

Author (year)	Resilience model	Operationalized definition of resilience
Quantitative, cross‐sectional or longitudinal studies (*n* = 35)		
Castellano‐Tejedor and Lusilla‐Palacios (2017)^58^	Hybrid (trait–process)	A range of thoughts, feelings and behaviours and a dynamic process encompassing positive adaptation within the context of significant adversity; it is also considered a personality characteristic that moderates the negative effects of stress and promotes adaptation
Senturk et al. (2018)^59^	Trait	The ability of a person to successfully overcome and adapt to negative conditions despite the difficult circumstances; satisfaction with social network and social support, psychological well‐being, strength and a healthy life
Garity (1997)^76^	Trait	A personality trait or characteristic that moderates the negative effects of stress and promotes adaptation; persons who display courage or adaptability in the face of adversity
Scholten et al. (2020)^60^	Trait	Psychological factor related to psychological distress
Brickell et al. (2020)^77^	Process	Core concepts of adversity and personal adaptation; the concept of personal adaptation allows for resilience to be a flexible rather than fixed process and may be modified over time as the individual adapts
Simpson and Jones (2013)^91^	Process	A multidimensional construct constituting a range of thoughts, feelings and behaviours; a dynamic process encompassing positive adaptation within the context of significant adversity
Cousins et al. (2013)^61^	Trait	The characteristic way in which people approach and cope with life events, described in terms of three related tendencies: commitment, where behaviour is influenced by the meaning and purpose seen in a situation; control, the ability to make one's own choices in a situation; and challenge, the tendency to perceive life events as opportunities for development, rather than threats
Elnasseh et al. (2016)^62^	Process	A psychological phenomenon characterized by effective coping and adaptation in the face of loss, hardship or adversity; a protective factor; and personal strength
Ertl et al. (2019)^63^	Trait	An individual's ability to adapt, persevere and maintain emotional equilibrium despite adversity; psychological strength
Fitzpatrick and Vacha‐Haase (2010)^78^	Hybrid (trait–process)	Resilient individuals are able to confront a crisis successfully and engage in positive behaviour to adjust coping strategies for effective adaptation to the situation; a multidimensional construct involving not only psychological traits but also the individual's ability to use external sources to facilitate coping
Kimura et al. (2019)^87^	Hybrid (trait–process)	A dynamic and complex construct that involves the interaction of both risk and protective factors, internal and external to the individual, that act to modify the effects of an adverse life event; a protective factor that enhances health by buffering the deleterious effects of stress
Ruisoto et al. (2020)^64^	Hybrid (trait–process)	A control‐related intrapsychic variable that may promote a more successful adaptation to care demands; personality trait, but broader approaches underline the importance of relational and situational contexts for resilience behaviour
Scott (2013)^46^	Process	A characteristic or developmental process in individuals that, when activated, aids in thwarting the effects of social conditions that can lead to impaired daily functioning
Pessotti et al. (2018)^88^	Trait	One's capacity for successful adaptation when faced with the stress of adversity; not invulnerability to stress, but, rather, the ability to recover from negative events
Wilks and Vonk (2008)^79^	Trait	Implies a track record of successful adaptation in the individual who has been exposed to stressful life events, and an expectation of continued low susceptibility to future stressors; reflects an outcome strength, that is, the ability to recover from the stressor successfully
Rosa et al. (2020)^90^	Trait	One's capacity for successful adaptation when faced with the stress of adversity
Chan et al. (2019)^83^	Trait	Successful adaptation and competence that results in effective functioning in the face of stressful situations
Dias et al. (2016)^89^	Trait	One's capacity for successful adaptation when faced with the stress of adversity; facilitates adaptation by enabling one to identify what is stressful, realistically appraise one's capacity for action and solve problems effectively; considered as a personality characteristic
Serra et al. (2018)^65^	Trait	The abilities and personal resources of individuals that allows them to successfully deal with adverse situations
Sutter et al. (2016)^47^	NR	Relates to positive coping strategies, lower depressive symptoms and positive psychosocial variables
Jones et al. (2018)^48^	Process	The process of adaptation to distress and is associated with the caregiver's ability to draw on personal assets in combination with the availability, suitability and use of community and societal resources
Jones, Killett et al. (2019a)^55^; Jones, Woodward et al. (2019b)^49^	Process	2019a
		Multidimensional concept that embodies personal qualities and external support systems that enable one to thrive in the face of adversity
	Process	2019b
		Positive adaptation to stressful situations and encompasses both individual characteristics and extrinsic factors, including social support from their family and the wider community
Wilks et al. (2011, 2018)^80^	Trait	2011
		Implies adaptational success; a characteristic of psychological well‐being, referring to the ability to recover from negative life events, leading to hope and expectation of success in the face of future adversity; reflects postadversity strength boosted by protective factors
	Trait	2018
		The positive role of the ubiquitous phenomenon of individual difference in people's responses to stress and adversity reflects an outcome of strength, recovery and hardiness postadversity
Wilks (2008a)^66^; Wilks and Croom (2008b)^82^	Trait	2008a
		An adaptational outcome success; suggests overcoming the odds, adapting to high risk (adversity) and recovering from adversity by adjusting successfully to negative life events
	Trait	2008b
		Viewed as being augmented by protective factors and defined as a psychological phenomenon referring to effective coping and adaptation although faced with loss, hardship or adversity
Anderson et al. (2019)^92^	Hybrid (trait–process)	The ability to adapt in the face of tragedy, trauma, adversity, hardship and ongoing significant life stressors; a multidimensional construct comprising a mix of personal skills and attributes, social competence, social resources and spirituality, which may be associated with reductions in morbidity and increased positive well‐being
Hayas et al. (2015)^50^	Process	The process of positive adaptation in the face of adversity, trauma, tragedy, threats or significant sources of stress; a dynamic process in which psychological, social, environmental and biological factors interact to enable an individual at any stage of life to develop, maintain or regain his or her mental health despite exposure to adversity
Vatter et al. (2018, 2020)^56^, ^57^	Trait	The ability to bounce back from stress
Ledbetter et al. (2020)^67^	Hybrid (trait–process)	An individual's successful adaption to adversity or stressful experiences, informed by both elements of their personality and the contextual, ongoing situation in which adversity occurs, but is frequently measured at a discrete point in time; a combination of both personality and situational factors that inform how individuals cope with stress and adversity
O'Rourke et al. (2010)^68^	Process	The process of adaptation in response to adversity, threats or significant stress such as the diagnosis and care of a family member with a major illness
Rivera‐Navarro et al. (2018)^51^	Trait	Protective factor
Tyler et al. (2020)^69^	Process	The process of negotiating, managing and adapting to significant sources of stress or trauma; assets and resources within the individual, their life and environment facilitate this capacity for adaptation and ‘bouncing back’ in the face of adversity
Quantitative, intervention studies (*n* = 4)		
Ghaffari et al. (2019)^84^	Process	Describes a situation in which a caregiver improves social performance and overcome difficulties, despite experiencing high mental pressure
Lavretsky et al. (2010)^75^	NR	NR
MacCourt et al. (2017)^70^	Trait	A positive personality characteristic that enhances individual adaptation, preserving balance and harmony
Pandya (2019)^85^	NR	NR
Mixed‐methods studies (*n* = 4)		
Maneewat et al. (2016)^86^	Process	A process of growth and adaption with a multidimensional structure; a holistic and dynamic development that encompasses the ability to cope with stress and serious situations
Bull (2014)^71^	Process	A dynamic process that fluctuates across time and situations and enables individuals to adjust or cope successfully despite stress or adversity
Jones et al. (2019)^54^	Process	A dynamic and interactive phenomenon, which is triggered by an antecedent event and developed through the interplay of risks and resources
Kidd et al. (2011)^72^	Trait	Human beings are engaged in goal‐directed movement that has unified patterns and utilizes creative power (resilience) to overcome obstacles; resilience is a positive psychological resource
Qualitative studies (*n* = 7)		
Bekhet and Avery (2018)^19^	Trait	When homoeostasis is restored after adversity, which includes new insight and growth from a disruptive experience
Roberts and Struckmeyer (2018)^73^	Hybrid (trait–process)	The ability to maintain normal or enhanced functioning during times of adversity and consists of two components: the first is thriving and succeeding; the second is showing the competence in difficult situations or a situation where others often do not succeed
Han et al. (2019)^25^	Trait	To be able to restore balance and harmony when they encounter negative circumstances, which may be achieved by enhancing inherent adaptation
Liu et al. (2020)^74^	Process	The process of effectively negotiating, adapting to or managing significant sources of stress or trauma; assets of and resources available to the individual, their life and environment facilitate this capacity for adaptation and ‘bouncing back’ in the face of adversity; across the life course, the experience of resilience will vary
Donnellan et al. (2015, 2017, 2019)^38^, ^52^, ^53^	Process	The process of effectively negotiating, adapting to or managing significant sources of stress or trauma; assets of and resources available to the individual, their life and environment facilitate this capacity for adaptation or bouncing back in the face of adversity

Abbreviation: NR, not reported.

Beyond these commonalities, researchers differed in terms of whether they defined resilience as a multidimensional process or a personality trait. On one side of this discordance, some studies (*n* = 8, 16%) used language that illustrated resilience as a personal quality, skill or attribute enabling caregivers to adapt in the experience of hardship.^49^, ^55^, ^64^, ^67^, ^70^, ^76^, ^78^, ^92^ Comparably, resilience was described in articles (*n* = 7, 14%) as an individual's ability or capacity to adjust successfully and maintain normal functioning despite adverse trauma,^63^, ^65^, ^73^, ^79^, ^80^, ^88^, ^89^ alluding to the belief that resilience is a fixed competence. In fact, the main aim of one study was to test the hypothesis that caregiver resilience is a personality trait, after which it was concluded that resilience is, indeed, an individual characteristic.^89^ Thus, a total of 22 (44%) studies explicitly advanced trait definitions of resilience.^19^, ^25^, ^51^, ^56^, ^57^, ^59^, ^60^, ^61^, ^63^, ^65^, ^66^, ^70^, ^72^, ^76^, ^79^, ^80^, ^81^, ^82^, ^83^, ^88^, ^89^, ^90^ In contrast, a substantial proportion (*n* = 18, 36%) of researchers opted for construing resilience as a dynamic process.^38^, ^46^, ^48^, ^49^, ^50^, ^52^, ^53^, ^54^, ^55^, ^62^, ^68^, ^69^, ^71^, ^74^, ^77^, ^84^, ^86^, ^91^ Some (*n* = 7, 14%) authors used a more mixed model of resilience, presenting the concept of resilience as a hybrid of both a personality characteristic and an evolving process.^58^, ^64^, ^67^, ^73^, ^78^, ^87^, ^92^

#### 3.2.2 Origins of resilience definitions

Authors conceptualized resilience in a diversity of forms, citing numerous sources in support of their interpretation. Many authors interpreted resilience from a combination of sources, electing to not advance a singular definition. Some studies (*n* = 12, 24%) specified the use of a specific resilience theory or framework.^19^, ^25^, ^38^, ^52^, ^53^, ^67^, ^76^, ^79^, ^80^, ^81^, ^83^, ^86^ Of the frameworks explicitly included, the Ecological Resilience Framework^31^ applied to caregivers was most common and incorporated into four studies (8%).^25^, ^38^, ^52^, ^53^ Two sets of original resilience theorists were frequently accredited as the primary source of authors' understanding and characterization of resilience. These theorists include (1) Wagnild and Young,^37^ who offer a trait‐based resilience definition, and (2) Windle et al.,^29^, ^31^ who conceptualize resilience as an unfixed process. The former was utilized in some studies (*n* = 4, 8%),^70^, ^76^, ^88^, ^90^ and the latter was found in multiple studies (*n* = 8, 16%).^38^, ^48^, ^52^, ^53^, ^54^, ^69^, ^74^, ^77^

#### 3.2.3 Measurement and levels of resilience

In quantitative and mixed‐methods studies, 12 different instruments were used to measure resilience. A summary of the 12 scales is presented in Table 4. Three studies (6%) used caregiver‐specific measurements.^50^, ^85^, ^86^ The most commonly used scale was the Resilience Scale (RS) by Wagnild and Young.^37^ Twelve studies (24%)^46^, ^58^, ^70^, ^71^, ^72^, ^76^, ^78^, ^87^, ^88^, ^89^, ^90^, ^91^ used the full version of the RS, whereas four studies (8%) used the short form.^66^, ^80^, ^81^, ^82^ A second common scale was the CD‐RISC.^32^, ^93^, ^94^ Seven studies (14%) used the full version of the CD‐RISC,^51^, ^64^, ^65^, ^75^, ^79^, ^84^, ^92^ while one (2%) study used a shortened form.^60^ Another commonly used scale was the six‐item BRS,^36^ reported in seven studies (14%).^47^, ^56^, ^57^, ^63^, ^67^, ^69^, ^83^ Original validation of the included scales reported acceptable to strong internal consistency (*α* = .67–.95). Several of the retained studies (18%) assessed resilience scale reliability within their caregiver samples,^46^, ^59^, ^62^, ^63^, ^67^, ^69^, ^84^, ^85^, ^87^ demonstrating acceptable to strong internal consistency (*α* = .73–.96). Three studies (6%) developed and validated instruments to measure caregiver resilience,^50^, ^66^, ^86^ and reported strong reliability (*α* = .87–.96).

**Table 4:** Description of resilience measures included in the review

Scale	Author(s)	Country of origin and language	Target population	Number of dimensions (items)	Cronbach's *α*	Retained studies that assessed scale reliability (CNC population)
The Connor–Davidson Resilience Scale	Connor and Davidson (2003)^32^	USA/English	Adults	5 (25)	.89–.93^a^ .87	Ghaffari et al. (2019)^84^ (AD)
The Connor–Davidson Resilience Scale (shortened version)	Cambell‐Sills and Stein (2007)^94^	USA/English	Young adults	1 (10)	.85^a^	
The Resilience Scale	Wagnild and Young (1993)^37^	Australia/English	Adults	2 (25)	.91^a^ .80 .94	Kimura et al. (2019)^87^ (YOAD) Scott (2013)^46^ (AD)
The Resilience Scale (shortened version of RS)	Neill and Dias (2001)^95^	Australia/English	Adults	14–15	.91^a^ .96	Wilks (2008)^66^ (AD)
The Brief Resilience Scale	Smith et al. (2008)^36^	USA/English	Adults	1 (6)	.80–.91^a^ .73 .82 .89	Ertl et al. (2019)^63^ (PD) Ledbetter et al. (2020)^67^ (SCI) Tyler et al. (2020)^69^ (PD)
The Resilience Scale for Adults	Friborg et al. (2003)^96^	Norway/Norwegian	Adults	5 (36)	.67–.90^a^	
The Resilience Scale for Adults	Friborg et al. (2005)^97^	Norway/Norwegian	Adults	6 (33)	.76–.87^a^ .96 .92 .82	Elnasseh et al. (2016)^62^ (dementia) Pandya (2019)^85^ (AD) Senturk et al. (2018)^59^ (dementia)
TBI‐QOL Resilience Short Form	Tulsky et al. (2016)^98^	USA/English	TBI	1 (27)	.95^a^	
The Dispositional Resilience Scale	Bartone et al. (1989)^99^	USA/English	Adults	3 (45)	.78^a^	
The Brief Resilient Coping Scale	Sinclair and Wallston (2004)^100^	USA/English	Adults with rheumatoid arthritis	1 (4)	.69^a^	
Questionnaire of Resilience in Caregivers of Acquired Brain Injury	Hayas et al. (2015)^50^	Spain/Spanish	Family caregivers of persons with ABI	1 (31)	.88^a^	Hayas et al. (2015)^50^ (ABI)^b^
The Caregiver Resilience Scale	Maneewat et al. (2016)^86^	Thailand/Thai	Family caregivers of persons with dementia	6 (30)	.87^a^ .87	Maneewat et al. (2016)^86^, b (dementia) Pandya (2019)^85^ (AD)

Abbreviations: ABI, acquired brain injury; AD, Alzheimer's disease; PD, Parkinson's disease; YOAD, young‐onset Alzheimer's disease.

^a^ Indicates the *α* value reported in the original scale development and validation.

^b^ Indicates original scale validation and article retained within current review.

Most quantitative and intervention studies assessed and reported the level of resilience among sampled caregivers. However, the majority of studies (*n* = 22, 44%) did not interpret these resilience measures in reference to scale‐based criteria or in comparison to other populations; instead, most attended to other resilience‐related results, incorporating resilience as a modulator, outcome or into higher‐level models. Within those that did measure and interpret resilience as a continuous variable, sampled caregivers demonstrated moderate‐to‐high‐resilience levels in seven articles (14%),^58^, ^71^, ^76^, ^82^, ^83^, ^87^, ^89^ while two articles (4%) also inferred low‐resilience levels in a minority of participants.^58^, ^71^ Numerous studies (*n* = 10, 20%) sought to categorically classify participants into different resilience groups, either as resilient or nonresilient,^38^, ^52^, ^53^ or in some version of low‐, medium‐ or high‐resilience groups.^49^, ^54^, ^55^, ^56^, ^57^, ^77^, ^91^

### 3.3 Correlates and predictors of psychological resilience

#### 3.3.1 Sociodemographics and contextual resources

As summarized in Table 5, 12 (24%) studies examined sociodemographic or contextual factors including gender, injury or disease severity and clinical symptoms associated with resilience.^48^, ^49^, ^57^, ^58^, ^60^, ^67^, ^77^, ^83^, ^87^, ^88^, ^89^, ^90^ Our synthesis revealed heterogeneity in studies reporting the relationship between sociodemographic and clinical characteristics and resilience. Six articles (12%) demonstrated that demographic, CNC severity, clinical and health status variables were not significantly related to caregivers' resilience levels.^58^, ^77^, ^87^, ^89^, ^90^, ^91^ In contrast, findings from 11 studies (22%) indicated that demographic and clinical variables were significantly related to resilience, including income,^62^, ^85^ employment status,^83^ gender,^49^, ^72^, ^83^, ^85^ ethnicity,^81^, ^85^ age^72^ and severity of dementia.^88^

**Table 5:** Quantitative, mixed‐methods and qualitative articles' descriptions and summaries of resilience findings

Author (year)	Purpose	Recruitment setting	Resilience scale or measure	Mean resilience score (SD)	Key results
Quantitative, cross‐sectional or longitudinal studies (*n* = 35)					
Castellano‐Tejedor and Lusilla‐Palacios (2017)^58^	To describe a sample of caregivers of persons SCI, their burden of care, resilience and life satisfaction and to assess the relationships between these variables and other sociodemographic factors	SCI acute unit from a tertiary university hospital following discharge	The Resilience Scale	141.93 (23.4)	Half of the sample showed moderate–high resilience; few had low‐resilience scores. Resilience was not related to caregivers' demographics or SCI severity. Burden was negatively correlated with resilience. Resilience was positively correlated with relationship satisfaction
Senturk et al. (2018)^59^	To examine the relationship between caregiver burden and psychological resilience in caregivers of PWD	Outpatient neurology department of a university hospital	The Resilience Scale for Adults	111.25 (23.9)	Negative correlation between the caregiver burden index and resilience scores
Garity (1997)^76^	To investigate the relationship between stress level, learning style, resilience factors and ways of coping in AD family caregivers	Support groups of an AD association	The Resilience Scale	144.4^c^	Participants were moderate–high on resilience scores and used problem‐ and emotion‐focused coping. Resilience positively correlated with emotion‐ and problem‐focused coping
Scholten et al. (2020)^60^	To identify intra‐ and interpersonal sociodemographic, injury‐related and psychological variables measured at admission of inpatient rehabilitation that predict psychological distress among dyads of individuals with SCI or ABI and their significant others 6 months after discharge	Part of a larger study conducted in regional rehabilitation centres	Connor–Davidson Resilience Scale Short‐form	28.2 (6.1)	Higher baseline psychological distress, lower scores on adaptive psychological characteristics (combination of self‐efficacy, proactive coping, purpose in life and resilience), and higher scores on maladaptive psychological characteristics (combination of passive coping, neuroticism, appraisals of threat and loss) were related to higher psychological distress, as well as crosswise between individuals with SCI or ABI and their significant others
Brickell et al. (2020)^77^	To examine factors related to resilience in military caregivers across health‐related caregiver QOL, caregiver sociodemographic variables and SMV injury and health status	TBI clinics at a National Military Medical Centre; Marine Corps base camp; community outreach activities	TBI‐QOL Resilience Short form	55.6 (9)	There were no differences across caregiver resilience groups (‘low‐moderate’, ‘moderate’, ‘moderate‐high’) for most demographics, SMV injury and health status variables. Low resilience was related to strain on employment due to caregiving duties, financial burden, caring for children, less personal time, caring for both verbal and physical irritability, anger and aggression and lower SMV functionality. Lower resilience was associated with poorer health‐related QOL scores across all groups
Simpson and Jones (2013)^91^	To investigate the relationship between resilience and positive affect, negative affect and burden in caregiving; the relationship between resilience and helpfulness of caregiving management strategies; and the similarities and differences in resilience among family TBI versus ABI caregivers	Review of medical records and staff caseloads	The Resilience Scale	140.2 (18.7)	Positive correlation between resilience and positive affect. Resilience demonstrated a negative correlation with negative affect and burden scores. No link was found between resilience and the relatives' severity of functional impairment. Participants with high‐resilience scores rated certain caregiving strategies as more helpful than those with low‐resilience scores
Cousins et al. (2013)^61^	To explore the influence of family caregivers on the uptake of NIV in persons with MND	Specialist neurology and respiratory clinics	The Dispositional Resilience Scale	NIV	Caregivers supporting NIV treatment were more resilient. Caregiver resilience (commitment) was the strongest predictor of uptake of NIV treatment
				88.63 (13.2)	
				Decliners	
				73.50 (15)	
Elnasseh et al. (2016)^62^	To examine whether healthier family dynamics are associated with a higher sense of coherence, resilience and optimism in dementia caregivers in Latin America	Regional Neuroscience Institute	The Resilience Scale for Adults	204.29 (21.8)	Family dynamics explained 32% of the variance in resilience. Income was associated with resilience. Greater family empathy and decreased family problems were associated with higher resilience
Ertl et al. (2019)^63^	To examine whether resilience moderates the relation between perceived stress and health‐related QOL among PD caregivers in Mexico	Outpatient neuropsychological services at the National Neuroscience Institute	The Brief Resilience Scale	21.28 (4.4)	Resilience moderated the inverse relationship between perceived stress and mental health‐related QOL. Resilience did not moderate the relation between stress and physical health‐related QOL
Fitzpatrick and Vacha‐Haase (2010)^78^	To examine the relationship between resilience and marital satisfaction in caregivers of spouses with dementia	Gerontology Research Unit at regional hospital and local caregiver support groups	The Shortened Resilience Scale	5.5 (0.8)	Resilience was not correlated with marital satisfaction. Marital satisfaction was influenced most by caregiver burden (negative influence) and caregiver age (positive influence)
Kimura et al. (2019)^87^	To investigate the relationship between clinical symptoms of people with young‐onset Alzheimer disease (YOAD) and carer resilience	AD outpatient clinic at the University Institute of Psychiatry	The Resilience Scale	141.4 (13.5)	Carers showed moderate to high levels of resilience. No relationship was found between carer resilience and both carer and care‐recipient sociodemographic characteristics. No relationship was found between career resilience and clinical symptoms of persons with YOAD. Resilience was inversely associated with carers' depressive symptoms
Ruisoto et al. (2020)^64^	To examine factors that predict burden in a sample of family caregivers of PWD	Referral lists of the associations of relatives of people with AD and other dementias, neurology outpatient clinics and the national reference centre of AD	The Connor‐Davidson Resilience Scale	73.9 (13.7)	Caregiver burden correlated negatively with resilience. Resilience explained 18.7% of variance in social support and social support accounted for 46.11% of variance in burden. Social support partially mediated the relationship between resilience and burden in caregivers
Scott (2013)^46^	To examine the moderating effect of resilience between caregiver stressors and caregiver burden	Community agencies that provide education and support to AD caregivers in the region	The Resilience Scale	NR	Resilience was not identified as a moderator of the relationship between stressors and caregiver burden. An inverse relationship existed between resilience and caregiver burden
Pessotti et al. (2018)^88^	To evaluate family caregivers' perception of QOL, burden, resilience and religiosity and relate them with cognitive aspects and occurrence of neuropsychiatric symptoms of elderly persons with dementia	Clinical Neurology Outpatient Clinic at the regional hospital	The Resilience Scale	135.6 (22.5)	Resilience was associated with better perceived QOL, severity of dementia, higher intrinsic religiosity and lower occurrence of depressive symptoms
Wilks and Vonk (2018)^79^	To explore whether the coping method of private prayer served as a protective factor or mediator between caregiver burden and perceived resiliency among AD caregivers	Regional AD association caregiver support groups	The Connor–Davidson Resilience Scale	73.4 (13.4)	Burden positively affected the extent of prayer usage and negatively influenced resilience. Caregiver burden and private prayer influenced variation in resilience scores. Results support prayer as a mediator between burden and resilience
Rosa et al. (2020)^90^	To investigate resilience in caregivers of people with mild and moderate AD and the related sociodemographic and clinical characteristics	Outpatient clinic of the university institute of psychiatry and AD	The Resilience Scale	140.6 (17.2)	In persons with mild and moderate AD, caregiver resilience was inversely related to emotional problems. There was no difference between resilience in caregivers of people with mild versus moderate AD. In the mild AD group, neuropsychiatric symptoms of the person with AD and caregiver's depressive symptoms were related to caregiver resilience. In the moderate AD group, caregiver QOL and coresiding with the care‐recipient were related to resilience
Chan et al. (2019)^83^	To explore caregiver strain and resilience of caregivers of patients with AD in Malaysia; to determine factors associated with caregiver strains in caregivers of patients with AD; and to determine the effect of resilience on the relationship between caregiver strains and caregiver or patient factors	AD Foundation Malaysia	The Brief Resilience Scale	19.2 (3.3)	The sample demonstrated moderate to high resilience. Resilience was associated with gender and employment status. A negative correlation was found between resilience and caregiver strain
Dias et al. (2016)^89^	To investigate the relationship between resilience and sociodemographic and clinical factors of people with dementia; to test the hypothesis that caregivers' resilience is a personality trait, independent from the clinical symptoms of the person with dementia	Physicians' referral from a dementia outpatient clinic	The Resilience Scale	137.6 (15.5)	Participants reported moderate to high levels of resilience. Resilience was not related to gender, clinical or emotional problems. Resilience was related to caregiver QOL, and inversely associated with depressive symptoms. There was no relationship between caregivers' resilience and sociodemographic and clinical characteristics of people with dementia. The authors concluded that resilience is an individual characteristic
Serra et al. (2018)^65^	To investigate a set of caregiver and patient factors, such as psychosocial protective variables, linked to abuse‐related behaviour of PWD	Referrals from the associations of relatives of PWD, neurology outpatient clinics and The National Reference Centre of AD	The Connor–Davidson Resilience Scale	73.9 (13.7)	Resilience and social support were negatively associated with abuse scores (i.e., protective effect). Social support and resilience were associated with a lower probability of abuse
Sutter et al. (2016)^47^	To examine the relationships between personal strengths (optimism, sense of coherence and resilience) and mental health of dementia caregivers from Latin America	Regional neuroscience institute and university, local neurology outpatient clinics, flyers, word‐of‐mouth, local community connections	The Brief Resilience Scale	17.4 (5.6)	More manageability, general resilience and social competence were uniquely associated with lower depression. Resilience and other variables were not predictive of caregiver burden or life satisfaction
Jones et al. (2018)^48^	To describe the demographic and psychosocial characteristics of caregivers who attend dementia cafes and to identify which factors influence the likelihood of family caregivers attending dementia cafes	Dementia cafes and health and well‐being events facilitated by local AD or well‐being societies	The Brief Resilient Coping Scale	NR	Caregivers who attended cafes reported higher resilience and subjective well‐being; no difference in social support was detected
Jones, Killett et al. (2019a)^55^; Jones, Woodward et al. (2019b)^49^	2019a	Adverts in newsletters, carer information events held by local charities and an online carer's forum, dementia cafes	The Brief Resilient Coping Scale	NR^a^	2019a
	To investigate factors that affect resilient coping in carers; to assess whether symptoms of distress vary between carers with differing levels of resilient coping; and to identify whether resilient coping acts as a mediator in the carer distress–well‐being relationship				‘High’ resilient carers reported less distress than ‘low’ resilient carers. Resilient coping partially mediated the relationships between well‐being and caregiver distress (i.e., depression, anxiety, stress and burden). Carers with high resilient coping skills reported less depression, anxiety, stress and burden than those with ‘low’ resilient coping
	2019b				2019b
	To compare sociodemographic characteristics and the availability of social support for carers with ‘low’ and ‘high’ resilient coping and to identify if social support predicted high resilient coping in informal carers of people with dementia				The availability of emotional/informational support was most likely to predict resilient coping and tangible support was the least likely to predict resilient coping. Only gender predicted high resilient coping. No single domain of social support had a greater influence on resilient coping
Wilks et al. (2011, 2018)^80^, ^81^)	2011	Mailing lists from a nonprofit AD services organisation; African American communities (e.g., churches, community centres, adult day centres, a home health agency, caregiver homes)	The Shortened Resilience Scale	2011	2011
	This study assessed the impact of AD patients' aggressive behaviour (i.e., AD aggression) on caregiver coping strategies (task‐, emotion‐, and avoidance‐focused) and caregiver resilience, and examined whether a coping strategy moderated the AD aggression–caregiver resilience relationship			5.9^c^	Aggression negatively predicted caregiver resilience. All coping strategies correlated with resilience scores. Task‐focused coping was positively related to resilience. Emotion and avoidance‐focused coping strategies separately interacted with aggression and increased their negative relationship with resilience. Task‐focused coping showed no moderating effect
	2018			2018	2018
	To understand whether spiritual support with AD caregivers acts as a moderating factor among the caregiving burden–resilience relationship in a manner similar to caregiver social support, and to observe ethnicity, African American versus Caucasian caregivers, in said moderation			5.8^c^	For each ethnic group of caregivers, burden was inversely proportional to resilience. In all groups, the association between spiritual support and resilience was positive and direct. Social support did not moderate risk within either group. African American caregivers reported higher resilience than their Caucasian counterparts
Wilks (2008a)^66^; Wilks and Croom (2008b)^82^	2008a	Two large AD care conferences: one held in a large urban area and another held in a rural locale	The Shortened Resilience Scale	5.5 (1.3)	2008a
	To evaluate psychometric properties of the shortened Resilience Scale among a sample of AD caregivers				Results confirmed the RS15 to be a psychometrically sound measure that can be used to appraise the efficacy of caregiving adaptability among the sample
	2008b				2008b
	To examine whether social support functions as a protective, resilience factor among AD caregivers; to examine the relationship between risk (i.e., perceived stress) to mental and physical health, an outcome of resilience and potential protective factors for resilience among caregivers				The sample reported moderate to high resilience. Perceived stress negatively influenced resilience and accounted for 43% of variance in resilience scores. Social support positively influenced resilience, and caregivers with high family support had the highest probability of elevated resilience. Social support is a protective mediator of resilience
Anderson et al. (2019)^92^	To integrate related explanatory (personality, coping) and mediating (hope, resilience, self‐efficacy) and caregiver outcome (burden, psychological distress, quality of life) variables into a larger model and to test the role of resilience, hope and self‐efficacy among family caregivers of persons with TBI	Six regional inpatient and community rehabilitation centres	The Connor–Davidson Resilience Scale	76.23 (12.3)	The model accounted for 63% of the variance in resilience. Resilience had a direct effect on positive affect in caregivers. There was a strong positive association between general self‐efficacy and resilience. Problem‐focused coping had a direct positive effect on resilience. Resilience was indirectly associated with caregiver burden when mediated through social support. Resilience demonstrated a direct effect on hope that is associated with positive mental health. Resilience was associated with reduced morbidity
Hayas et al. (2015)^50^	To develop the Questionnaire of Resilience in Caregivers of Acquired Brain Injury (QRC‐ABI) and explore its psychometric properties	The Federation of ABI Associations and public day care centres specializing in ABI	QRC‐ABI	43.24 (11.3)	The QRC‐ABI showed good reliability and validity. Convergent validity was supported through positive correlations of the QRC‐ABI with QOL, positive aspects of caregiving and posttraumatic growth and a negative correlation with perceived burden
Vatter et al. (2018, 2020)^56^, ^57^	2018	Nation‐wide post or as part of a larger study (ref)	The Brief Resilience Scale	24.97 (11.9)	2018
	To explore the factor structure of the Zarit Burden Interview (ZBI) in life partners of people with Parkinson's‐related dementia and to examine the relationships among the emerging factors and the demographic and clinical features				Five factors of the ZBI (i.e., social and psychological constraints, personal strain, interference with personal life, concerns about future and guilt) all negatively correlated with resilience. Lower resilience and higher negative strain and feelings of resentment were contributors to burden
	2020				2020
	To explore and compare levels of mental health, care burden and relationship satisfaction among caregiving spouses of people with mild cognitive impairment or dementia in PD (PDD) or dementia with Lewy bodies (DLB)				Over 75% of respondents reported good resilience. ZBI scores correlated with resilience. Caregivers who were dissatisfied with their relationship reported lower resilience. Burden, stress, resilience, relationship satisfaction, quality of life, anxiety, depression and mental health levels did not differ between spouses of people with PDD and DLB.
Ledbetter et al. (2020)^67^	To investigate how individual and contextual factors (i.e., caregiving tasks, resilience, timing of the SCI) moderate the extent to which receiving social support predicts psychosocial distress among SCI caregiving romantic partners	Online groups targeted at SCI caregivers	The Brief Resilience Scale	4.05 (0.8)	Resilience inversely predicted psychosocial distress in both the preinjury and postinjury groups. Findings revealed the benefits of resilience. Receiving high‐quality support and timing of the injury moderated resilience effects. Injuries sustained after relationship initiation threatened well‐being and closeness and altered the extent to which support and resilience were associated with health and relationship benefits
O'Rourke et al. (2010)^68^	To examine the three facets of psychological resilience (i.e., perceived control, commitment to living, challenge versus stability) as predictors of depressive symptoms over time among spousal caregivers of PwAD	Clinic for AD and related disorders at a regional university hospital	The Dispositional Resilience Scale	NR	Resilience was associated with depressive symptoms among caregivers. Challenge and perceived control predicted depressive symptoms 1 year later. An increase in challenges over time predicted lower levels of depressive symptoms at Time 2. Commitment was not associated with depressive symptoms at any time point
Rivera‐Navarro et al. (2018)^51^	To validate the Caregiver Abuse Screen (CASE) as an instrument for detecting the maltreatment of people with dementia in Spain	Local associations of relatives of people with AD and other dementia and neurology outpatient clinics	The Connor–Davidson Resilience Scale	73.6 (13.4)	High CASE scores were associated with greater burden, lower social support and lower resilience of caregivers. Resilience scores were negatively correlated with interpersonal abuse and neglect/dependency. The consistent negative association of CASE scores with resilience is indicative of this advantageous characteristic
Tyler et al. (2020)^69^	To validate a theoretical structural equation model whereby social support is associated with higher levels of resilience in PD caregivers and increased resilience is related to decreased mental health symptoms	PD clinics associated with academic university institutions in Mexico and the PD and Movement Disorders Center at a regional medical centre in the USA	The Brief Resilience Scale	NR	The model explained 11% of the variance in resilience. Higher levels of social support were associated with higher resilience, which in turn was associated with lower mental health symptoms. Resilience partially mediated the effect of social support on mental health symptoms
Quantitative, intervention studies (*n* = 4)					
Ghaffari et al. (2019)^84^	To determine the effectiveness of resilience education in the mental health of family caregivers of elderly patients with AD	Referrals from regional hospital and neurologist offices	The Connor–Davidson Resilience Scale	NR	Resilience education promoted the mental health of family AD caregivers by decreasing somatic symptoms and social dysfunction
Lavretsky et al. (2010)^75^	To examine the potential of an antidepressant drug (escitalopram) to improve depression, resilience to stress and quality of life in family dementia caregivers in a randomized placebo‐controlled double‐blinded trial	NR	The Connor–Davidson Resilience Scale	60.2 (16.7)	Measures of depression, anxiety, resilience, burden and distress and quality of life improved on escitalopram compared with placebo groups
MacCourt et al. (2017)^70^	To assess the structure and effectiveness of a grief management coaching intervention with caregivers of individuals with dementia	Local social media and referrals from regional AD society	The Resilience Scale	Spouse T1: 67.9^c^ T2: 68.9^c^	For the intervention group, grief, coping, empowerment and resilience scores improved postintervention. The intervention group showed greater resilience at Time 2. Time 1 resilience scores predicted greater resilience at Time 2
				Adult child T1: 66.6^c^ T2: 71.1^c^	
Pandya (2019)^85^	To report the impact of a long‐term meditation programme for enhancing self‐efficacy and resilience of home‐based caregivers of older adults with AD	Network of agencies linked to older adults, geriatric clinics and units in private hospitals	The Resilience Scale for Adults; The Caregiver Resilience Scale (CRS)	RSA Control Pre: 99.2 (8.3) Post: 100 (8.3) Intervention Pre: 100.31 (9) Post:187.93 (14.2)	Posttest RSA and CRS scores of the intervention group were higher than the control group and their own pretest scores. Caregiver women, spouses, Hindus, middle class, with college and higher education, homemakers, who attended at least 75% of the meditation lessons and regularly practiced meditation at home reported lower posttest perceived caregiving burden, higher self‐efficacy and resilience. Meditation was effective for increasing resilience
				CRS Control Pre: 30.28 (4.4) Post: 31.03 (5.3) Intervention Pre: 31.21 (4.9) Post: 58.71 (6.8)	
Mixed‐methods studies (*n* = 4)					
Maneewat et al. (2016)^86^	To develop the CRS for Thai caregivers of older persons with dementia and to examine its validity and reliability	Memory Clinic, Neurological Clinic or Geriatric Clinic in the Outpatient Department at a regional hospital	The CRS; semi‐structured interviews	NR	The final version of the CRS was composed of 30 items within six domains: physical competence; relationship competence; emotional competence; cognitive competence; moral competence; and spiritual competence. The 30‐item CRS was considered a valid and reliable instrument
Bull (2014)^71^	To describe family caregivers' level of resilience and psychological distress and to describe the strategies that family caregivers use to persevere in their caregiving role despite the challenges encountered in caring for a family member with dementia	Five adult day centres located in a city setting	The Resilience Scale; narrative interviews	154.3 (15.8)	Participants had high resilience and low psychological distress. The use of self‐sustaining strategies explained the high scores on resilience and low levels of psychological distress. Caregivers used four strategies to sustain the self: drawing on past life experiences that dealt with difficult situations, nourishing the self, relying on spirituality and seeking dementia‐related information
Jones et al. (2019)^54^	To explore discrepancies and congruency between definitions of resilience in the academic literature and carers' own conceptualizations; to assess differences and similarities in conceptualizations of resilience between carers with high‐, medium‐ and low‐resilience scores; and to compare carers' perceived level of resilience with the level of resilience when measured on a standardized tool	Theoretical sampling recruited from participants in previous study^48^	Brief Resilient Coping Scale; semi‐structured interviews	NR	Under half (46%) of the carers had low resilience. Carers' definitions of resilience were concordant with clinical and academic definitions; however, they extended the concept and placed greater value on the role of self‐compassion. Carers recognized that the appearance of resilience may have negative consequences in terms of securing support from others. Resilience scores did not always match carers' own perceptions of their level of resilience
Kidd et al. (2011)^72^	To test the effectiveness of a poetry writing intervention for family caregivers of elders with dementia and to examine outcome variables of self‐transcendence, resilience, depressive symptoms and subjective caregiver burden	Support groups, churches and agencies	The Resilience Scale; interviews	NR	Women were lower in self‐transcendence and resilience, and higher in depressive symptoms and burden. Older caregivers scored higher than younger caregivers on the study variables of self‐transcendence and resilience. Poetry writing was an effective intervention that may promote resilient outcomes

Qualitative studies (*n* = 7)					
**Author (year)**	**Purpose**	**Recruitment setting**	**Means of resilience assessment**	**Key results**	
Bekhet and Avery (2018)^19^	To identify components of resilience theory (i.e., risk factors, protective factors, overlapping factors) from the perspective of caregivers of PWD	Regional AD Association early stage programmes	Open‐ended questions on written questionnaires	The experience of dementia caregiving involved a combination of risk factors and protective factors, suggesting that caregivers may feel conflicted. Risk factors included experiences of stress and difficulties, demanding tasks, frustration, lack of social support, exhaustion and negative feelings. Protective factors included feeling rewarded and serving a purpose. If protective factors were more predominant, then caregivers became more resilient and experienced associated positive health outcomes	
Roberts and Struckmeyer (2018)^73^	To examine family caregiver perspectives on how respite programming impacts their resilience and ability to better handle the demands of their responsibilities	Recruited as part of a larger study^b^ through respite providers	Semi‐structured interviews	Several themes emerged describing the path to caregiver resilience that included family dynamics, isolation, financial struggles, seeking respite and acceptance. The road to acceptance became a critical factor in the development of resilience	
Han et al. (2019)^25^	To identify challenges, possible solutions as resources for resilience and expected consequences from the perspective of family caregivers of hospice patients with dementia	Two large hospice agencies recruited as part of a larger clinical trial	Deductive content analysis of secondary clinical trial data	Resilience resources were identified at the individual, community and societal levels. Resources included knowledge, self‐control and appraisal, self‐care, using visual materials, having options to choose a good care facility with exemplary providers, family or friends' support, involvement in volunteer activities, legislative support, public awareness and health insurance. Identified challenges were difficulties in communication, providing care and decision‐making, lack of knowledge, emotional challenges, concern about care facility selection, death with dignity and lack of public awareness	
Liu et al. (2020)^74^	To investigate the resilience of a growing but largely underserved and understudied population—Chinese American dementia caregivers, whose experience is embedded in their development throughout the life span, process of migration and sociocultural contexts	Local agency providing services for dementia caregivers with a representation of Chinese clients	Semi‐structured interviews	Main themes fit within two categories, challenge and resilience, in each of the four principles—time and place, timing in lives, linked lives and agency—of the developmental life course perspective. Physical and emotional exhaustion was the most frequently mentioned challenge theme, followed by limited knowledge of dementia, navigating the healthcare system and limited time for self‐development. Three aspects of resilience—sense of mastery, access to formal and informal support and commitment to care—were salient among caregivers	
Donnellan et al. (2015, 2017, 2019)^38^, ^52^, ^53^	2015	Two local dementia support groups and a care home	Semi‐structured interviews	2015	
	To assess whether spousal dementia carers can achieve resilience and to reveal which factors and resources facilitate or hinder resilience within the ecological framework			Carers achieve resilience via a complex multidimensional process. A resilient carer was someone who stayed positive, who maintained their relationship with their loved one's former self, who were knowledgeable, well supported and who were engaged with respite services. Facilitating community factors included friendships with common experience and social participation. Individual hindering resilience factors were negative outlook and perceived social isolation	
	2017			2017	
	To explore social support as a key component of resilience and to identify the availability and function of support provided to older spousal dementia carers			Social support is not always sufficient to facilitate resilience, as negative perceptions of support may moderate the effect of support on resilience. Family and friends served a wide range of functions, but were equally available to resilient and nonresilient participants	
	2019			2019	
	To use qualitative longitudinal methods to examine trajectories of resilience and which assets and resources are associated with resilience and care status transitions in spousal dementia carers			Five participants remained resilient, three remained nonresilient and four participants became resilient. Only one participant became nonresilient. Stable resilience was characterized by continuing individual assets and community resources. Carers who became resilient returned to previous resources or gained new resources	

Abbreviations: ABI, acquired brain injury; AD, Alzheimer's disease; CRS, caregiver resilience scale; MND, motor neuron disease; NIV, noninvasive ventilation; NR, not reported; PD, Parkinson's disease; PWD, persons with dementia; QOL, quality of life; RSA, resilience scale for adults; SCI, spinal cord injury; SMV, service member veteran; TBI, traumatic brain injury.

^a^ Authors divided participants into ‘low’ (BRCS 0–13), ‘medium’ (BRCS 14‐16) and ‘high’ (BRCS 17+) resilient groups, with no inclusion of the total mean resilience scores.

^b^ Reference for a large study not provided.

^c^ SD for resilience scores not reported.

Three studies (6%) investigated resilience resources or assets—markers that are typically positioned conceptually upstream to resilience development. For instance, studies broadly examined resilience resources at multiple ecological levels.^25^, ^38^, ^53^ In three explorative articles (6%), resilience resources emerged at the individual, community and societal levels.^25^, ^38^, ^53^ In five cases (10%), resilience was related to specific behaviours, such as treatment uptake,^61^, ^75^ private prayer^79^ and likelihood of care recipient abuse or neglect.^51^, ^65^

### 3.4 Social support and relational outcomes

Social support availability was a predominant construct assessed as an antecedent to resilience.^49^, ^52^, ^65^, ^67^, ^69^, ^82^ Among three studies (6%), social support was repeatedly predictive of resilience,^49^, ^69^, ^82^ with emotional and informational support the most likely to predict resilience. Specifically, four studies (8%) examined the association between resilience and relationship outcomes, including relationship satisfaction^56^, ^67^, ^78^ and family dynamics.^62^ Three studies (6%) determined that resilience was linked to romantic relationship benefits,^67^ relationship satisfaction^56^ and family dynamics (e.g., empathy, family problems).^62^ However, another study did not find resilience to be significantly correlated with marital satisfaction.^78^

### 3.5 Caregiver burden

A pattern across study objectives emerged, such that resilience was conceptually explored as a protective factor in opposition to caregiver burden. Indeed, numerous studies (*n* = 13, 26%) sought to examine the relationship between psychological resilience and burden.^46^, ^56^, ^57^, ^58^, ^59^, ^64^, ^72^, ^79^, ^81^, ^83^, ^88^, ^91^, ^92^ A key finding congruous across 12 reviewed studies (24%) was that caregiver burden was inversely associated with resilience.^46^, ^54^, ^56^, ^57^, ^58^, ^59^, ^64^, ^79^, ^81^, ^83^, ^91^, ^92^ Occasionally, caregiver burden was investigated in tandem with other variables reflective of well‐being such as life satisfaction,^58^ positive or negative affect,^91^ social support,^64^, ^81^ general distress,^46^, ^72^ quality of life^88^ and coping. Within seven studies (14%) that evaluated the association between resilience and burden, resilience was significantly and positively linked to multiple well‐being outcomes, namely, relationship satisfaction,^58^ positive affect,^91^, ^92^ social or spiritual support,^51^, ^81^ quality of life^88^ and coping.^92^ In fact, in one recent study, social support mediated the relationship between resilience and burden.^64^

### 3.6 General health outcomes

Independently of burden, resilience was explored in association with a number of positive and negative health outcomes; these variables consisted of psychological distress,^54^, ^60^, ^63^, ^67^, ^71^, ^75^, ^76^, ^82^ health‐related quality of life,^63^, ^75^, ^77^ mental health^47^, ^68^, ^69^, ^70^, ^84^ and coping strategies.^76^, ^80^ Three studies (6%) that examined coping and its connection with resilience determined that coping strategies correlated with resilience,^76^, ^80^, ^92^ specifically problem‐focused coping^76^, ^92^ and emotion‐focused coping.^76^ A second notable result among six studies (12%) was the inverse relationship between resilience and psychological distress,^54^, ^60^, ^63^, ^67^, ^71^, ^82^ reinforcing the concept that resilience assumes an adaptive psychological function and attenuates stress. Similarly, findings from 15 studies (30%) that did not involve burden showed that resilience exerted a positive impact on mental health outcomes, with a persistent inverse association with depressive symptoms,^47^, ^54^, ^68^, ^72^, ^87^, ^88^, ^89^, ^90^ and a direct association with quality of life,^63^, ^77^, ^88^, ^89^ along with other general mental health indicators.^19^, ^70^, ^84^

## 4 DISCUSSION

We undertook this review to document the scope of published research on psychological resilience among informal family caregivers of adults with CNCs. The volume of reviewed studies published within the last ten years is evidence that resilience is being increasingly investigated in the caregiving field, particularly within the context of dementia. However, this increased interest in resilience is accompanied by minimal conceptual consensus from a mosaic of scholarly origins, and findings suggest a lingering debate between process‐ and trait‐based definitions.

Some popular measurement tools were detected in included studies (i.e., RS, CD‐RISC, BRS), and yet, a plethora of scales were used to assess self‐perceived resilience, with minimal use of caregiver‐specific instruments despite characteristic similarities. Furthermore, in reports of resilience levels, some interpreted resilience as a continuous variable, while others categorically divided samples into stratified high–low resilient groups, making it difficult to judge how resilience capacities compared to other populations. A broad array of resilience predictors and correlates were observed, whereby resilience was inversely associated with burden, distress and depressive symptoms, and directly associated with various caregiver well‐being indicators including quality of life, coping, social support and mental health. These results confirm the potential for resilience to be leveraged within caregiver health promotion initiatives via policy and public practice.

Retained articles advanced a range of definitions of resilience. Although most resilience descriptions converged on components of adaptation and healthy functioning, the range of conflicting conceptualizations demonstrates that any form of consensus regarding resilience in family caregiver research has yet to be achieved. On one end of dissent, there was prevailing fixation on individual resilience, with conceptualization and measurement of resilience as a fixed characteristic or personality trait. These definitions appeared in sharp contrast to contemporary understandings of resilience as a dynamic process that interacts with the surrounding environment. Disagreement surrounding how caregiver resilience is defined and operationalized, in addition to the lack of a widely accepted theory or conceptual framework, renders investigation of the construct inconsistent.^101^

One retained study explored conceptual resilience discrepancies between academic definitions of resilience and caregivers' personal conceptualizations, and found that caregivers extended the concept more broadly and emphasized the role of self‐compassion.^54^ The implications of self‐compassion as a protective factor for psychological wellness have been previously documented in family caregivers,^102^ and may represent a useful avenue for future resilience research. With this study^54^ as an important example of a more caregiver‐centred approach, researchers should strive to actively involve vulnerable informal caregiver populations within the research process, commencing with resilience conceptualization and moving towards harmonization with clinical and academic definitions.

Similarly, the lack of a persistent definition of resilience within caregiver health research perpetuates the tendency of scholars to position resilience within statistical models as either a modulator of well‐being or a binary outcome itself that is either present or absent.^27^ In actuality, modern resilience researchers argue that resilience is likely to exist on a continuum that fluctuates across different domains and the life course.^29^ Nevertheless, conceptual inconsistencies hinder the validity and generalisability of resilience findings and represent a barrier to providing direction for the development of clinical applications designed to enhance resilience within targeted caregiver populations. To mitigate these inconsistencies moving forward, CNC caregiving research should attempt to follow a unified caregiver‐centred resilience framework to navigate this robust interdisciplinary construct across developmental trajectories.

The current divergence in resilience conceptualization and assessments may be representative of the relative novelty of this concept in comparison to other long‐standing psychological constructs that have had been comprehensively validated across populations and widespread contexts. Equivalently, the differing use of quantitative resilience scales supports the notion that there is no gold standard of resilience assessment.^33^ The absence of homogeneity in resilience measurement undermines the ability of researchers, clinicians and community members to reliably monitor and evaluate the efficacy of resilience‐building programmes.^103^ This lack of standardization further prevents resilience levels from being compared across different caregiver subpopulations.^27^ Similarly, with few developed and validated condition‐specific scales within the resilience field, it is difficult to reliably verify and contrast resilience levels within distinct caregiving populations. It is recommended that future resilience and caregiving research draw on contemporary views of resilience from broad literature to formulate context‐specific measures, while attending to the evolving theory and research. This will surely elevate the quality of resilience‐based research moving forward, while preventing further dispersion within the field.

The underrepresentation of caregivers of persons with less common CNCs in the included studies was apparent and presents a challenge for advancing disease‐specific resilience applications. Few studies examined sudden‐onset conditions, there was an absence of stable and intermittent types of conditions and progressive conditions consisted of mostly dementia. As we approach a saturation of research in resilience in dementia caregiving, resilience in caregiving populations of other CNCs (e.g., MS, MND, epilepsy, cerebral palsy) remains understudied. This is problematic because it has been empirically proven that caregiver demographics and health outcomes vary as a function of the specific CNC encountered.^104^ It is important that such overlooked populations garner further interest in the field of resilience investigation, allowing their respective resilience processes to be equitably understood, measured and harnessed.

To account for discrepancies in the caregiving experience across different CNCs, there remains dispute within the literature concerning the degree of influence that demographic and clinical factors exert on resilience processes. The studies reviewed herein captured the relationship between resilience and age, gender, income, employment status, ethnicity and clinical injury or disease severity; some studies reported that these factors were significantly associated with resilience,^49^, ^62^, ^72^, ^81^, ^83^, ^85^, ^88^ while others did not.^58^, ^77^, ^87^, ^89^, ^90^, ^91^ This debate is further compounded by the fact that most sampled caregivers were spousal and middle‐aged women from homogeneous cultural regions. This dispute clouds current understanding of the intersection between resilience and individual and contextual factors related to the caregiver population, such as biological underpinnings, environment and culture—all of which have been integrated into resilience perspectives.^27^, ^105^, ^106^ In line with socioecological models of resilience,^31^, ^105^, ^107^ it is imperative to clarify which sociodemographic and environmental factors facilitate resilience development in CNC caregivers via a culturally sensitive approach that embraces heterogeneity.^41^ One way to determine how contextual factors influence the resilience trajectory is by conducting longitudinal studies.^27^ As most of the included articles (80%) originated from western English‐speaking countries, multicultural representation within resilience and caregiver research remains deficient. Future research should strive to explore CNC caregiver resilience across cultures to accurately profile differing environmental and demographic factors and their influence on resilience within diverse community settings.

Finally, we observed resilience exploration in connection to caregiver burden in one quarter of the included studies. This prevalence suggests that resilience research in CNC family caregivers is inconsistent with emergent resilience research, as broader disciplines now favour a strengths‐ and competence‐based approach.^26^, ^27^ We acknowledge that a proportion of the reviewed studies (30.5%) excluded burden from their design and depicted the positive link between resilience and well‐being. However, it appears that many researchers continue to examine the negative consequences of caregiving, and how resilience protects caregivers from impending risk innate to their role. This, in turn, fails to abandon the outmoded deficit‐based model of caregiver resilience and mental health.^27^ Despite the expected inverse relationship between resilience and burden, we caution against the assumption that caregiver burden and resilience can coexist, such that one determines the other. Instead, it is advised to position each concept as mutually exclusive, as the exact causal mechanisms responsible for their association remain unknown.^100^, ^105^ Arguably, through conflation of dimensions of flourishing and languishing, our understanding of resilience becomes obscured by the pathologies and dysfunction denoted by caregiver burden.^107^ It is suggested that scholars adopt a more proactive or preventative approach that prioritizes building strengths,^27^ while simultaneously no longer assuming that CNC caregiving is uniformly burdensome.

### 4.1 Limitations

This study had some limitations. First, resilience is a wide‐ranging and nuanced concept that parallels with other psychological topics (e.g., hardiness, adaptation, coping) across psychosocial disciplines. To address this concern, we adopted a broad approach in our initial search strategy to acquire as much relevant literature as possible; still, it is possible that we missed relevant literature because of our resilience‐specific focus. Second, due to the nature of many variables often examined in close association with resilience, it is possible that there was potential methodological bias among the retained studies in describing these relationships, including issues of simultaneity and reverse causality.

There is scarcity of research in family caregivers of more uncommon conditions (e.g., MS, MND, epilepsy) that limited the extent to which the protective role of resilience in more unique care‐providing situations could be explored. We are cautious about offering condition‐specific conclusions at this time because of the overrepresentation of dementia‐related caregiver populations, and the overall heterogeneity of the included studies. Lastly, this study was limited by its inclusion of only English‐language publications, which limited our search to studies conducted primarily in North America and Europe. This limitation made it challenging to obtain diverse geographical representation and assess how resilience varies widely across countries and cultures.

## 5 CONCLUSION

This review synthesized existing knowledge of resilience in family caregiving for persons living with a CNC. Findings revealed an insufficient level of agreement among researchers with respect to how resilience is theorized, conceptualized and assessed. This emphasizes the fact that resilience is a complex, multifaceted phenomenon that merits further clarification within the caregiving sphere with respect to whether it is a trait, process or a hybrid of the two. Collective findings demonstrate that resilience is associated with better overall health and psychological well‐being, and contributes to optimal stress management among CNC family caregivers. The ideal context in which resilience develops and how that process varies cross‐culturally has yet to be determined, though this represents a useful direction for future research and complements newfound socio‐ecological resilience theories. Furthermore, while a strengths‐based approach does not currently unanimously prevail across the reviewed literature, there is room for evolution to dissociate weakness‐, risk‐ and deficit‐focused models of caregiving from resilience and to cultivate approaches rooted in caregiver empowerment. With limited representation of intervention studies, there is a need to develop targeted interventions for informal CNC caregivers aimed at promoting resilience and increase awareness of the positive aspects of caregiving.

## AUTHOR CONTRIBUTIONS

The underlying search strategy was developed by the first author (Odessa McKenna), second author (Afolasade Fakolade), third author (Katherine Cardwell) and the corresponding author (Lara A. Pilutti). The search strategy was refined, conducted and managed by the fourth author (Nigèle Langlois). The corresponding author (Lara A. Pilutti) merged results into the appropriate data management software and removed duplicates. Together, the first (Odessa McKenna), third (Katherine Cardwell), and fifth (Karen Jiang) authors completed the article screening process. The first (Odessa McKenna) and third (Katherine Cardwell) authors completed data extraction. This manuscript, including the introduction, results, discussion and conclusion sections was written by the first author (Odessa McKenna), while the methods section was written in collaboration with the fourth author (Nigèle Langlois). This manuscript was edited extensively by authors Afolasade Fakolade and Lara A. Pilutti. Authors Karen Jiang and Katherine Cardwell contributed moderately to final revisions.

## Supporting information

Supporting information.

Supporting information.

## Data Availability

Data sharing is not applicable to this article as no new data were created or analysed in this study.

## References

[1] Ouriques Martins SC , Sacks C , Hacke W , et al. Global, regional, and national burden of neurological disorders, 1990–2016: a systematic analysis for the Global Burden of Disease Study 2016. Lancet Neurol. 2019;18(5):459‐480. 10.1016/S1474-4422(18)30499-X 30879893 PMC6459001

[2] Turner‐Stokes L , Sykes N , Silber E . Long‐term neurological conditions: management at the interface between neurology, rehabilitation and palliative care. Clin Med. 2008;8(2):186‐191. 10.7861/clinmedicine.8-2-186 PMC4953006 18478869

[3] Turner‐Stokes L , Sykes N , Silber E , Khatri A , Sutton L , Young E . From diagnosis to death: exploring the interface between neurology, rehabilitation and palliative care in managing people with long‐term neurological conditions. Clin Med. 2007;7(2):129‐136. 10.7861/clinmedicine.7-2-129 PMC4951826 17491500

[4] Simmons RD , Tribe KL , McDonald EA . Living with multiple sclerosis: longitudinal changes in employment and the importance of symptom management. J Neurol. 2010;257(6):926‐936. 10.1007/s00415-009-5441-7 20084515

[5] Terriff DL , Williams JVA , Patten SB , Lavorato DH , Bulloch AGM . Patterns of disability, care needs, and quality of life of people with Parkinson's disease in a general population sample. Park Relat Disord. 2012;18(7):828‐832. 10.1016/j.parkreldis.2012.03.026 22542396

[6] Fisk JD , Pontefract A , Ritvo PG , Archibald CJ , Murray T . The impact of fatigue on patients with multiple sclerosis. Can J Neurol Sci. 1994;21(1):9‐14. 10.1111/ane.13244 8180914

[7] McKenzie T , Quig ME , Tyry T , et al. Care partners and multiple sclerosis: differential effect on men and women. Int J MS Care. 2015;17(6):253‐260. 10.7224/1537-2073.2014-083 26664330 PMC4673917

[8] Hlabangana V , Hearn JH . Depression in partner caregivers of people with neurological conditions; associations with self‐compassion and quality of life. J Ment Health. 2020;29(2):176‐181. 10.1080/09638237.2019.1630724 31241383

[9] McKeown LP , Porter‐Armstrong AP , Baxter GD . The needs and experiences of caregivers of individuals with multiple sclerosis: a systematic review. Clin Rehabil. 2003;17(3):234‐248. 10.1191/0269215503cr618oa 12735530

[10] Hassan A , Wu SS , Schmidt P , et al. What are the issues facing Parkinson's disease patients at ten years of disease and beyond? Data from the NPF‐QII study. Park Relat Disord. 2012;18(Suppl 3):14‐18. 10.1016/j.parkreldis.2012.06.014 22776044

[11] Greenwood N , Smith R . Barriers and facilitators for male carers in accessing formal and informal support: a systematic review. Maturitas. 2015;82(2):162‐169. 10.1016/j.maturitas.2015.07.013 26271710

[12] Ma M , Dorstyn D , Ward L , Prentice S . Alzheimers' disease and caregiving: a meta‐analytic review comparing the mental health of primary carers to controls. Aging Ment Health. 2018;22(11):1395‐1405. 10.1080/13607863.2017.1370689 28871796

[13] Legg L , Weir CJ , Langhorne P , Smith LN , Stott DJ . Is informal caregiving independently associated with poor health? A population‐based study. J Epidemiol Community Health. 2013;67(1):95‐97. 10.1136/jech-2012-201652 22875077

[14] Figved N , Myhr KM , Larsen JP , Aarsland D . Caregiver burden in multiple sclerosis: the impact of neuropsychiatric symptoms. J Neurol Neurosurg Psychiatry. 2007;78(10):1097‐1102. 10.1136/jnnp.2006.104216 17237144 PMC2117557

[15] Martínez‐Martín P , Forjaz MJ , Frades‐Payo B , et al. Caregiver burden in Parkinson's disease. Mov Disord. 2007;22(7):924‐931. 10.1002/mds.21355 17238193

[16] Berg A , Palomäki H , Lönnqvist J , Lehtihalmes M , Kaste M . Depression among caregivers of stroke survivors. Stroke. 2005;36(3):639‐643. 10.1161/01.STR.0000155690.04697.c0 15677575

[17] Gupta S , Goren A , Phillips AL , Stewart M . Self‐reported burden among caregivers of patients with multiple sclerosis. Int J MS Care. 2012;14(4):179‐187. 10.7224/1537-2073-14.4.179 24453750 PMC3882984

[18] Eska K , Graessel E , Donath C , Schwarzkopf L , Lauterberg J , Holle R . Predictors of institutionalization of dementia patients in mild and moderate stages: a 4‐year prospective analysis. Dement Geriatr Cogn Dis Extra. 2013;3(1):426‐445. 10.1159/000355079 24348504 PMC3843910

[19] Bekhet AK , Avery JS . Resilience from the perspectives of caregivers of persons with dementia. Arch Psychiatr Nurs. 2018;32(1):19‐23. 10.1016/j.apnu.2017.09.008 29413066

[20] Pakenham KI . The positive impact of multiple sclerosis (MS) on carers: associations between carer benefit finding and positive and negative adjustment domains. Disabil Rehabil. 2005;27(17):985‐997. 10.1080/09638280500052583 16096252

[21] Delle Fave A , Bassi M , Allegri B , et al. Beyond disease: happiness, goals, and meanings among persons with multiple sclerosis and their caregivers. Front Psychol. 2017;8:1‐15. 10.3389/fpsyg.2017.02216 29326635 PMC5742493

[22] Cohen CA , Colantonio A , Vernich L . Positive aspects of caregiving: rounding out the caregiver experience. Int J Geriatr Psychiatry. 2002;17(2):184‐188. 10.1002/gps.561 11813283

[23] Mackenzie A , Greenwood N . Positive experiences of caregiving in stroke: a systematic review. Disabil Rehabil. 2012;34(17):1413‐1422. 10.3109/09638288.2011.650307 22309576

[24] Luthar SS , Cicchetti D , Becker B . The construct of resilience: a critical evaluation and guidelines for future work. Child Dev. 2000;71(3):543‐562. 10.1111/1467-8624.00164 10953923 PMC1885202

[25] Han S , Chi NC , Han C , Oliver DP , Washington K , Demiris G . Adapting the resilience framework for family caregivers of hospice patients with dementia. Am J Alzheimers Dis Other Demen. 2019;34(6):399‐411. 10.1177/1533317519862095 31364381 PMC7179812

[26] Fergus S , Zimmerman MA . Adolescent resilience: a framework for understanding healthy development in the face of risk. Annu Rev Public Health. 2005;26:399‐419. 10.1146/annurev.publhealth.26.021304.144357 15760295

[27] Southwick SM , Bonanno GA , Masten AS , Panter‐Brick C , Yehuda R . Resilience definitions, theory, and challenges: interdisciplinary perspectives. Eur J Psychotraumatol. 2014;5:5. 10.3402/ejpt.v5.25338 PMC4185134 25317257

[28] Ong AD , Bergeman CS , Bisconti TL , Wallace KA . Psychological resilience, positive emotions, and successful adaptation to stress in later life. J Pers Soc Psychol. 2006;91(4):730‐749. 10.1037/0022-3514.91.4.730 17014296

[29] Windle G . What is resilience? A review and concept analysis. Rev Clin Gerontol. 2011;21(2):152‐169. 10.1017/S0959259810000420

[30] Sullivan KA , Kempe CB , Edmed SL , Bonanno GA . Resilience and other possible outcomes after mild traumatic brain injury: a systematic review. Neuropsychol Rev. 2016;26(2):173‐185. 10.1007/s11065-016-9317-1 27154289

[31] Windle G , Bennett K . Caring relationships: how to promote resilience in challenging times. In: Ungar M , ed. The Social Ecology of Resilience: A Handbook of Theory and Practice. New York: Springer; 2011:219‐231.

[32] Connor KM , Davidson JRT . Development of a new Resilience Scale: the Connor‐Davidson Resilience Scale (CD‐RISC). Depress Anxiety. 2003;18(2):76‐82. 10.1002/da.10113 12964174

[33] Windle G , Bennett KM , Noyes J . A methodological review of resilience measurement scales. Health Qual Life Outcomes. 2011;9(1):2‐18. 10.1186/1477-7525-9-8 21294858 PMC3042897

[34] Zhou Y , Ishado E , O′Hara A , Borson S , Sadak T . Developing a unifying model of resilience in dementia caregiving: a scoping review and content analysis. J Appl Gerontol. 2020;40(10):1377‐1388. 10.1177/0733464820923549 32500766

[35] Hjemdal O , Friborg O , Braun S , Kempenaers C , Linkowski P , Fossion P . The Resilience Scale for Adults: construct validity and measurement in a Belgian sample. Int J Test. 2011;11(1):53‐70. 10.1080/15305058.2010.508570

[36] Smith BW , Dalen J , Wiggins K , Tooley E , Christopher P , Bernard J . The Brief Resilience Scale: assessing the ability to bounce back. Int J Behav Med. 2008;15(3):194‐200. 10.1080/10705500802222972 18696313

[37] Wagnild GM , Young HM . Development and psychometric evaluation of the Resilience Scale. J Nurs Meas. 1993;1(2):165‐178. 7850498

[38] Donnellan WJ , Bennett KM , Soulsby LK . What are the factors that facilitate or hinder resilience in older spousal dementia carers? A qualitative study. Aging Ment Health. 2015;19(10):932‐939. 10.1080/13607863.2014.977771 25410637

[39] Bennett KM , Reyes‐Rodriguez MF , Altamar P , Soulsby LK . Resilience amongst older Colombians living in poverty: an ecological approach. J Cross Cult Gerontol. 2016;31(4):385‐407. 10.1007/s10823-016-9303-3 27585577 PMC5110598

[40] O′Dwyer ST , Moyle W , Taylor T , Creese J , Zimmer‐Gembeck M . In their own words: how family carers of people with dementia understand resilience. Behav Sci. 2017;7(3):22‐24. 10.3390/bs7030057 PMC5618065 28825686

[41] Teahan Á , Lafferty A , McAuliffe E , et al. Resilience in family caregiving for people with dementia: a systematic review. Int J Geriatr Psychiatry. 2018;33(12):1582‐1595. 10.1002/gps.4972 30230018

[42] Palacio GC , Krikorian A , Gómez‐Romero MJ , Limonero JT . Resilience in caregivers: a systematic review. Am J Hosp Palliat Med. 2020;37(8):648‐658. 10.1177/1049909119893977 31830813

[43] Moher D , Shamseer L , Clarke M , et al. Preferred Reporting Items for Systematic Review and Meta‐Analysis Protocols (PRISMA‐P) 2015 statement. Syst Rev. 2015;4(1):2‐9. 10.1186/2046-4053-4-1 25554246 PMC4320440

[44] McGowan J , Sampson M , Salzwedel DM , Cogo E , Foerster V , Lefebvre C . PRESS peer review of electronic search strategies: 2015 guideline statement. J Clin Epidemiol. 2016;75:40‐46. 10.1016/j.jclinepi.2016.01.021 27005575

[45] Larkin J , Foley L , Smith SM , Harrington P , Clyne B . The experience of financial burden for people with multimorbidity: a systematic review of qualitative research. Health Expect. 2020;24:282‐295. 10.1111/hex.13166 33264478 PMC8077119

[46] Scott CB . Alzheimer's disease caregiver burden: does resilience matter? J Hum Behav Soc Environ. 2013;23(8):879‐892. 10.1080/10911359.2013.803451

[47] Sutter M , Perrin PB , Peralta SV , et al. Beyond strain: personal strengths and mental health of Mexican and Argentinean dementia caregivers. J Transcult Nurs. 2016;27(4):376‐384. 10.1177/1043659615573081 25712148

[48] Jones SM , Killett A , Mioshi E . What factors predict family caregivers' attendance at dementia cafés? J Alzheimers Dis. 2018;64(4):1337‐1345. 10.3233/JAD-180377 29991136

[49] Jones SM , Woodward M , Mioshi E . Social support and high resilient coping in carers of people with dementia. Geriatr Nurs. 2019;40(6):584‐589. 10.1016/j.gerinurse.2019.05.011 31178232

[50] Hayas CL , Arroyabe EL , Calvete E . Resilience in family caregivers of persons with acquired brain injury. Rehabil Psychol. 2015;60(3):295‐302. 10.1037/rep0000040 26009779

[51] Rivera‐Navarro J , Sepúlveda R , Contador I , et al. Detection of maltreatment of people with dementia in Spain: usefulness of the Caregiver Abuse Screen (CASE). Eur J Ageing. 2018;15(1):87‐99. 10.1007/s10433-017-0427-2 29531518 PMC5840088

[52] Donnellan WJ , Bennett KM , Soulsby LK . Family close but friends closer: exploring social support and resilience in older spousal dementia carers. Aging Ment Health. 2017;21(11):1222‐1228. 10.1080/13607863.2016.1209734 27438380

[53] Donnellan WJ , Bennett KM , Soulsby LK . How does carer resilience change over time and care status? A qualitative longitudinal study. Aging Ment Health. 2019;23(11):1510‐1516. 10.1080/13607863.2018.1503998 30449140

[54] Jones SM , Mioshi E , Killett A . Coping but not allowing the coping to be everything: resilience in informal dementia care. Health Soc Care Community. 2019;27(4):e289‐e297. 10.1111/hsc.12732 30844124

[55] Jones SM , Killett A , Eneida M . The role of resilient coping in dementia carers' wellbeing. Br J Neurosci Nurs. 2019;15(1):6‐12. 10.12968/bjnn.2019.15.1.6

[56] Vatter S , Stanmore E , Clare L , McDonald KR , McCormick SA , Leroi I . Care burden and mental ill health in spouses of people with Parkinson disease dementia and lewy body dementia. J Geriatr Psychiatry Neurol. 2020;33(1):3‐14. 10.1177/0891988719853043 31146617

[57] Vatter S , McDonald KR , Stanmore E , Clare L , Leroi I . Multidimensional care burden in Parkinson‐related dementia. J Geriatr Psychiatry Neurol. 2018;31(6):319‐328. 10.1177/0891988718802104 30244631

[58] Castellano‐Tejedor C , Lusilla‐Palacios P . A study of burden of care and its correlates among family members supporting relatives and loved ones with traumatic spinal cord injuries. Clin Rehabil. 2017;31(7):948‐956. 10.1177/0269215517709330 28637391

[59] Senturk SG , Akyol MA , Kucukguclu O . The relationship between caregiver burden and psychological resilience in caregivers of individuals with dementia. Int J Caring Sci. 2018;11(2):1223‐1230.

[60] Scholten EWM , Ketelaar M , Visser‐Meily JMA , Roels EH , Kouwenhoven M , Post MWM . Prediction of psychological distress among persons with spinal cord injury or acquired brain injury and their significant others. Arch Phys Med Rehabil. 2020;101(12):2093‐2102. 10.1016/j.apmr.2020.05.023 32599061

[61] Cousins R , Ando H , Thornton E , Chakrabarti B , Angus R , Young C . Determinants of accepting non‐invasive ventilation treatment in motor neurone disease: a quantitative analysis at point of need. Health Psychol Behav Med. 2013;1(1):47‐58. 10.1080/21642850.2013.848169 25264500 PMC4164238

[62] Elnasseh AG , Trujillo MA , Peralta SV , et al. Family dynamics and personal strengths among dementia caregivers in Argentina. Int J Alzheimers Dis. 2016;2016:2386728. 10.1155/2016/2386728 27413574 PMC4931077

[63] Ertl MM , Trapp SK , González Arredondo S , Rodríguez Agudelo Y , Arango‐Lasprilla JC . Perceived stress, resilience, and health‐related quality of life among Parkinson's disease caregivers in Mexico. Health Soc Care Community. 2019;27(5):1303‐1310. 10.1111/hsc.12767 31149757

[64] Ruisoto P , Contador I , Fernández‐Calvo B , et al. Mediating effect of social support on the relationship between resilience and burden in caregivers of people with dementia. Arch Gerontol Geriatr. 2020;86:103952. 10.1016/j.archger.2019.103952 31542631

[65] Serra L , Contador I , Fernández‐Calvo B , et al. Resilience and social support as protective factors against abuse of patients with dementia: a study on family caregivers. Int J Geriatr Psychiatry. 2018;33(8):1132‐1138. 10.1002/gps.4905 29797350

[66] Wilks SE . Psychometric evaluation of the Shortened Resilience Scale among Alzheimer's caregivers. Am J Alzheimers Dis Other Demen. 2008;23(2):143‐149. 10.1177/1533317507313012 18198238 PMC10846258

[67] Ledbetter AM , Carr K , Lynn G . When a romantic partner has a spinal cord injury: caregiving tasks and resilience as moderators of support quality on psychosocial distress and relational closeness. J Soc Pers Relat. 2020;37(8‐9):2551‐2577. 10.1177/0265407520929761

[68] O′Rourke N , Kupferschmidt AL , Claxton A , Smith JZ , Chappell N , Beattie BL . Psychological resilience predicts depressive symptoms among spouses of persons with Alzheimer disease over time. Aging Ment Health. 2010;14(8):984‐993. 10.1080/13607863.2010.501063 21069604

[69] Tyler CM , Henry RS , Perrin PB , et al. Structural equation modeling of Parkinson's caregiver social support, resilience, and mental health: a strength‐based perspective. Neurol Res Int. 2020;2020: 7906547. 10.1155/2020/7906547 32110449 PMC7042552

[70] Maccourt P , McLennan M , Somers S , Krawczyk M . Effectiveness of a grief intervention for caregivers of people with dementia. Omega. 2017;75(3):230‐247. 10.1177/0030222816652802 28701116

[71] Bull MJ . Strategies for sustaining self used by family caregivers for older adults with dementia. J Holist Nurs. 2014;32(2):127‐135. 10.1177/0898010113509724 24227181

[72] Kidd LI , Zauszniewski JA , Morris DL . Benefits of a poetry writing intervention for family caregivers of elders with dementia. Issues Ment Health Nurs. 2011;32(9):598‐604. 10.3109/01612840.2011.576801 21859411

[73] Roberts E , Struckmeyer KM . The impact of respite programming on caregiver resilience in dementia care: a qualitative examination of family caregiver perspectives. Inquiry. 2018;55:1–11. 10.1177/0046958017751507 PMC5808833 29424252

[74] Liu J , Lou Y , Wu B , Yuk‐Sim Mui AC . “I've been always strong to conquer any suffering:” challenges and resilience of Chinese American dementia caregivers in a life course perspective. Aging Ment Health. 2020:25(9):1‐9. 10.1080/13607863.2020.1793900 PMC7855650 32687392

[75] Lavretsky H , Siddarth P , Irwin MR . Improving depression and enhancing resilience in family dementia caregivers: a pilot randomized placebo‐controlled trial of escitalopram. Am J Geriatr Psychiatry. 2010;18(2):154‐162. 10.1097/JGP.0b013e3181beab1e 20104071 PMC2813456

[76] Garity J . Stress, learning style, resilience factors, and ways of coping in Alzheimer family caregivers. Am J Alzheimers Dis Other Demen. 1997;12(4):171‐178. 10.1177/153331759701200405

[77] Brickell TA , Wright MM , Lippa SM , et al. Resilience is associated with health‐related quality of life in caregivers of service members and veterans following traumatic brain injury. Qual Life Res. 2020;29(10):2781‐2792. 10.1007/s11136-020-02529-y 32500241

[78] Fitzpatrick KE , Vacha‐Haase T . Marital satisfaction and resilience in caregivers of spouses with dementia. Clin Gerontol. 2010;33(3):165‐180. 10.1080/07317111003776547

[79] Wilks S , Vonk ME . Private prayer among Alzheimer's caregivers: mediating burden and resiliency. J Gerontol Soc Work. 2008;50(3‐4):113‐131. 10.1300/J083v50n3_09 18510194

[80] Wilks SE , Little KG , Gough HR , Spurlock WJ . Alzheimer's aggression: influences on caregiver coping and resilience. J Gerontol Soc Work. 2011;54(3):260‐275. 10.1080/01634372.2010.544531 21462058

[81] Wilks SE , Spurlock WR , Brown SC , Teegen BC , Geiger JR . Examining spiritual support among African American and Caucasian Alzheimer's caregivers: a risk and resilience study. Geriatr Nurs. 2018;39(6):663‐668. 10.1016/j.gerinurse.2018.05.002 29807671

[82] Wilks SE , Croom B . Perceived stress and resilience in Alzheimer's disease caregivers: testing moderation and mediation models of social support. Aging Ment Health. 2008;12(3):357‐365. 10.1080/13607860801933323 18728949

[83] Chan EWL , Yap PS , Khalaf ZF . Factors associated with high strain in caregivers of Alzheimer's disease (AD) in Malaysia. Geriatr Nurs. 2019;40(4):380‐385. 10.1016/j.gerinurse.2018.12.009 30765175

[84] Ghaffari F , Rostami M , Fotokian Z , Hajiahmadi M . Effectiveness of resilience education in the mental health of family caregivers of elderly patients with Alzheimer's disease. Iran J Psychiatry Behav Sci. 2019;13(3):e69507. 10.5812/ijpbs.69507

[85] Pandya SP . Meditation program enhances self‐efficacy and resilience of home‐based caregivers of older adults with Alzheimer's: a five‐year follow‐up study in two South Asian cities. J Gerontol Soc Work. 2019;62(6):663‐681. 10.1080/01634372.2019.1642278 31314712

[86] Maneewat T , Lertmaharit S , Tangwongchai S . Development of Caregiver Resilience Scale (CRS) for Thai caregivers of older persons with dementia. Cogent Med. 2016;3(1):1257409. 10.1080/2331205x.2016.1257409

[87] Kimura NRS , Neto JPS , Santos RL , et al. Resilience in carers of people with young‐onset Alzheimer disease. J Geriatr Psychiatry Neurol. 2019;32(2):59‐67. 10.1177/0891988718824039 30651027

[88] Pessotti CFC , Fonseca LC , Maria G , Souza DA , Laloni DT . Family caregivers of elderly with dementia‐Relationship between religiosity, resilience, quality of life and burden. Dement Neuropsychol. 2018;12(4):408‐414. 10.1590/1980-57642018dn12-040011 30546852 PMC6289474

[89] Dias R , Simões‐Neto JP , Santos RL , et al. Caregivers' resilience is independent from the clinical symptoms of dementia. Arq Neuropsiquiatr. 2016;74(12):967‐973. 10.1590/0004-282x20160162 27991993

[90] Rosa R , Simões‐Neto JP , Santos RL , et al. Caregivers' resilience in mild and moderate Alzheimer's disease. Aging Ment Health. 2020;24(2):250‐258. 10.1080/13607863.2018.1533520 30499333

[91] Simpson G , Jones K . How important is resilience among family members supporting relatives with traumatic brain injury or spinal cord injury? Clin Rehabil. 2013;27(4):367‐377. 10.1177/0269215512457961 23012693

[92] Anderson MI , Daher M , Simpson GK . A predictive model of resilience among family caregivers supporting relatives with traumatic brain injury (TBI): a structural equation modelling approach. Neuropsychol Rehabil. 2019;30(10):1925‐1946. 10.1080/09602011.2019.1620787 31132931

[93] Vaishnavi S , Connor K , Davidson JRT . An abbreviated version of the Connor‐Davidson Resilience Scale (CD‐RISC), the CD‐RISC2: psychometric properties and applications in psychopharmacological trials. Psychiatry Res. 2007;152(2‐3):293‐297. 10.1016/j.psychres.2007.01.006 17459488 PMC2041449

[94] Campbell‐Sills L , Stein MB . Psychometric analysis and refinement of the connor–davidson resilience scale (CD‐RISC): Validation of a 10‐item measure of resilience. J Trauma Stress. 2007;20:(6):1019–1028. 10.1002/jts.20271 18157881

[95] Neill JT , Dias KL . Adventure Education and Resilience: The Double‐Edged Sword. J Adventure Educ Outdoor Learn. 2001;1(2):35–42. 10.1080/14729670185200061

[96] Friborg O , Hjemdal O , Rosenvinge JH , Martinussen M . A new rating scale for adult resilience: what are the central protective resources behind healthy adjustment? Int. J. Methods Psychiatr. Res. 2003;12:(2):65–76. 10.1002/mpr.143 12830300 PMC6878238

[97] Friborg O , Barlaug D , Martinussen M , Rosenvinge JH , Hjemdal O . Resilience in relation to personality and intelligence. Int. J. Methods Psychiatr. Res. 2005;14:(1):29–42. 10.1002/mpr.15 16097398 PMC6878482

[98] Tulsky DS , Kisala PA , Victorson D , et al. TBI‐QOL: Development and Calibration of Item Banks to Measure Patient Reported Outcomes Following Traumatic Brain Injury. J Head Trauma Rehabil. 2016;31:(1):40–51. 10.1097/htr.0000000000000131 25931184 PMC4697960

[99] Bartone P , Ursano R , Wright K , Ingraham L . The impact of military air disaster on the health of assistance workers: A prospective study. J Nerv Ment Dis. 1989;177(6):317–328. 10.1097/00005053-198906000-00001 2723619

[100] Sinclair VG , Wallston KA . The Development and Psychometric Evaluation of the Brief Resilient Coping Scale. Assessment. 2004;11:(1):94–101. 10.1177/1073191103258144 14994958

[101] Foster K , Roche M , Delgado C , Cuzzillo C , Giandinoto JA , Furness T . Resilience and mental health nursing: an integrative review of international literature. Int J Ment Health Nurs. 2019;28(1):71‐85. 10.1111/inm.12548 30294937

[102] Xu S , Zhang H , Wang J . Caregiver burden and depression among Chinese family caregivers: the role of self‐compassion. Mindfulness. 2020;11(7):1647‐1654. 10.1007/s12671-020-01378-7

[103] Serfilippi E , Ramnath G . Resilience measurement and conceptual frameworks: a review of the literature. Ann Public Coop Econ. 2018;89(4):645‐664. 10.1111/apce.12202

[104] LaVela SL , Landers K , Etingen B , Karalius VP , Miskevics S . Factors related to caregiving for individuals with spinal cord injury compared to caregiving for individuals with other neurologic conditions. J Spinal Cord Med. 2015;38(4):505‐514. 10.1179/2045772314Y.0000000240 24993244 PMC4612206

[105] Ungar M . The social ecology of resilience: addressing contextual and cultural ambiguity of a nascent construct. Am J Orthopsychiatry. 2011;81(1):1‐17. 10.1111/j.1939-0025.2010.01067.x 21219271

[106] Nemeth DG , Capps CM , Каппс КМ . Resilience: a cognitive and psychosocial phenomenon. Lurian J. 2021;2(1):80‐94. 10.15826/Lurian.2021.2.1.5

[107] Ungar M . Resilience, trauma, context, and culture. Trauma, Violence, Abuse. 2013;14(3):255‐266. 10.1177/1524838013487805 23645297

