# Supplementary material for: Towards conceptual convergence: A systematic review of psychological resilience in family caregivers of persons living with chronic neurological conditions
Source: Health Expect. 2021 Oct 22;25(1):4–37. doi: 10.1111/hex.13374 (PMC8849377; doi:10.1111/hex.13374)
Supplement: Supplementary file 1 — Supporting information. [file HEX-25-4-s001.docx]

**Appendix 1**

**Ovid MEDLINE(R) ALL <1946 to July 24, 2020>**

Search history sorted by search number ascending

| **#** | **Searches** | **Results** | **Type** |
| --- | --- | --- | --- |
| 1 | exp Dementia/ | 165450 | Advanced |
| 2 | dementia*.ti,ab. | 108724 | Advanced |
| 3 | alzheimer*.ti,ab. | 145080 | Advanced |
| 4 | exp Epilepsy/ | 111335 | Advanced |
| 5 | epilep*.ti,ab. | 136236 | Advanced |
| 6 | seizure disorder*.ti,ab. | 3825 | Advanced |
| 7 | Headache Disorders/ | 2339 | Advanced |
| 8 | (headache* adj3 disorder*).ti,ab. | 3854 | Advanced |
| 9 | migraine*.ti,ab. | 34642 | Advanced |
| 10 | exp Multiple Sclerosis/ | 58749 | Advanced |
| 11 | multiple sclerosis.ti,ab. | 74395 | Advanced |
| 12 | exp Parkinsonian Disorders/ | 80433 | Advanced |
| 13 | parkinson* disease*.ti,ab. | 92687 | Advanced |
| 14 | exp Stroke/ | 134533 | Advanced |
| 15 | stroke*.ti,ab. | 247120 | Advanced |
| 16 | exp Brain Injuries, Traumatic/ | 14562 | Advanced |
| 17 | (trauma* adj3 brain injur*).ti,ab. | 36593 | Advanced |
| 18 | concussion*.ti,ab. | 8249 | Advanced |
| 19 | brain damage, chronic/ or brain injury, chronic/ | 13989 | Advanced |
| 20 | (brain adj2 (damage* or injur*) adj2 chronic*).ti,ab. | 608 | Advanced |
| 21 | Amyotrophic Lateral Sclerosis/ | 18856 | Advanced |
| 22 | Amyotrophic Lateral Sclerosis.ti,ab. | 23016 | Advanced |
| 23 | ("Gehrig* Disease" or "Lou Gehrig* Disease").ti,ab. | 133 | Advanced |
| 24 | exp Brain Neoplasms/ | 151466 | Advanced |
| 25 | (brain adj2 (tumo?r* or neoplasm* or cancer*)).ti,ab. | 48231 | Advanced |
| 26 | Intracranial Neoplasm*.ti,ab. | 1139 | Advanced |
| 27 | Cerebral Palsy/ | 20582 | Advanced |
| 28 | cerebral pals*.ti,ab. | 22469 | Advanced |
| 29 | Dystonia/ | 6362 | Advanced |
| 30 | dystonia*.ti,ab. | 14827 | Advanced |
| 31 | huntington disease/ | 12087 | Advanced |
| 32 | huntington* disease*.ti,ab. | 15451 | Advanced |
| 33 | tourette syndrome/ | 4302 | Advanced |
| 34 | (tourette* adj2 (disorder* or syndrome*)).ti,ab. | 4984 | Advanced |
| 35 | exp Hydrocephalus/ | 23496 | Advanced |
| 36 | Hydrocepha*.ti,ab. | 26363 | Advanced |
| 37 | exp Muscular Dystrophies/ | 26642 | Advanced |
| 38 | (Muscular adj2 Dystroph*).ti,ab. | 23625 | Advanced |
| 39 | exp Spinal Cord Injuries/ | 48630 | Advanced |
| 40 | (spinal adj2 (cord or column) adj3 (trauma* or injur* or transection* or laceration* or contusion*)).ti,ab. | 42875 | Advanced |
| 41 | exp Spinal Dysraphism/ | 8269 | Advanced |
| 42 | spina bifida.ti,ab. | 7040 | Advanced |
| 43 | (malnutrition and neurologic* disorder*).ti,ab. | 89 | Advanced |
| 44 | (chronic adj3 pain adj3 neurologic* disorder*).ti,ab. | 33 | Advanced |
| 45 | neuroinfection*.ti,ab. | 497 | Advanced |
| 46 | or/1-45 | 1278513 | Advanced |
| 47 | exp Adaptation, Psychological/ | 126933 | Advanced |
| 48 | Resilience, Psychological/ | 5732 | Advanced |
| 49 | social adjustment/ | 23254 | Advanced |
| 50 | (resilienc* or hardiness or empowerment or rebound*).ti,ab. | 54539 | Advanced |
| 51 | ((healthy or positive) adj2 function*).ti,ab. | 7908 | Advanced |
| 52 | (psychological adj2 (resilienc* or adaptation or wellbeing)).ti,ab. | 2672 | Advanced |
| 53 | ((coping or adaptive) adj2 (skill* or behavio?r*)).ti,ab. | 11774 | Advanced |
| 54 | or/47-53 | 212699 | Advanced |
| 55 | marriage/ or parents/ or spouses/ | 94296 | Advanced |
| 56 | (spous* or "domestic partner*" or partner* or wife or wives or husband? or "significant other*" or marriage or marital or married).ti,ab. | 257691 | Advanced |
| 57 | or | 543565 | Advanced |
| 58 | Caregivers/ | 36638 | Advanced |
| 59 | (caregiv* or care-giv*).ti,ab. | 73074 | Advanced |
| 60 | carer*.ti,ab. | 14612 | Advanced |
| 61 | or/55-60 | 865609 | Advanced |
| 62 | 46 and 54 and 61 | 3616 | Advanced |
| 63 | exp animals/ not humans.sh. | 4720580 | Advanced |
| 64 | 62 not 63 | 3615 | Advanced |
